# Recent Developments in the Synthesis of β-Diketones

**DOI:** 10.3390/ph14101043

**Published:** 2021-10-13

**Authors:** Gonzalo de Gonzalo, Andrés R. Alcántara

**Affiliations:** 1 Organic Chemistry Department, University of Sevilla, c/Profesor García González 2, 41012 Sevilla, Spain; 2 Department of Chemistry in Pharmaceutical Sciences, Faculty of Pharmacy, Complutense University of Madrid, Plaza de Ramón y Cajal, s/n., 28040 Madrid, Spain

**Keywords:** 1,3-diketones, biological activity, organic synthesis, Claisen condensation, biocatalysis, organocatalysis, metal-based catalysis, hydration, oxidation, decarboxylative coupling

## Abstract

Apart from being one of the most important intermediates in chemical synthesis, broadly used in the formation of C–C bonds among other processes, the β-dicarbonyl structure is present in a huge number of biologically and pharmaceutically active compounds. In fact, mainly derived from the well-known antioxidant capability associated with the corresponding enol tautomer, β-diketones are valuable compounds in the treatment of many pathological disorders, such as cardiovascular and liver diseases, hypertension, obesity, diabetes, neurological disorders, inflammation, skin diseases, fibrosis, or arthritis; therefore, the synthesis of these structures is an area of overwhelming interest for organic chemists. This paper is devoted to the advances achieved in the last ten years for the preparation of 1,3-diketones, using different chemical (Claisen, hydration of alkynones, decarboxylative coupling) or catalytic (biocatalysis, organocatalytic, metal-based catalysis) methodologies: Additionally, the preparation of branched β-dicarbonyl compounds by means of α-functionalization of non-substituted 1,3-diketones are also discussed.

## 1. Introduction

**Scheme 1:**
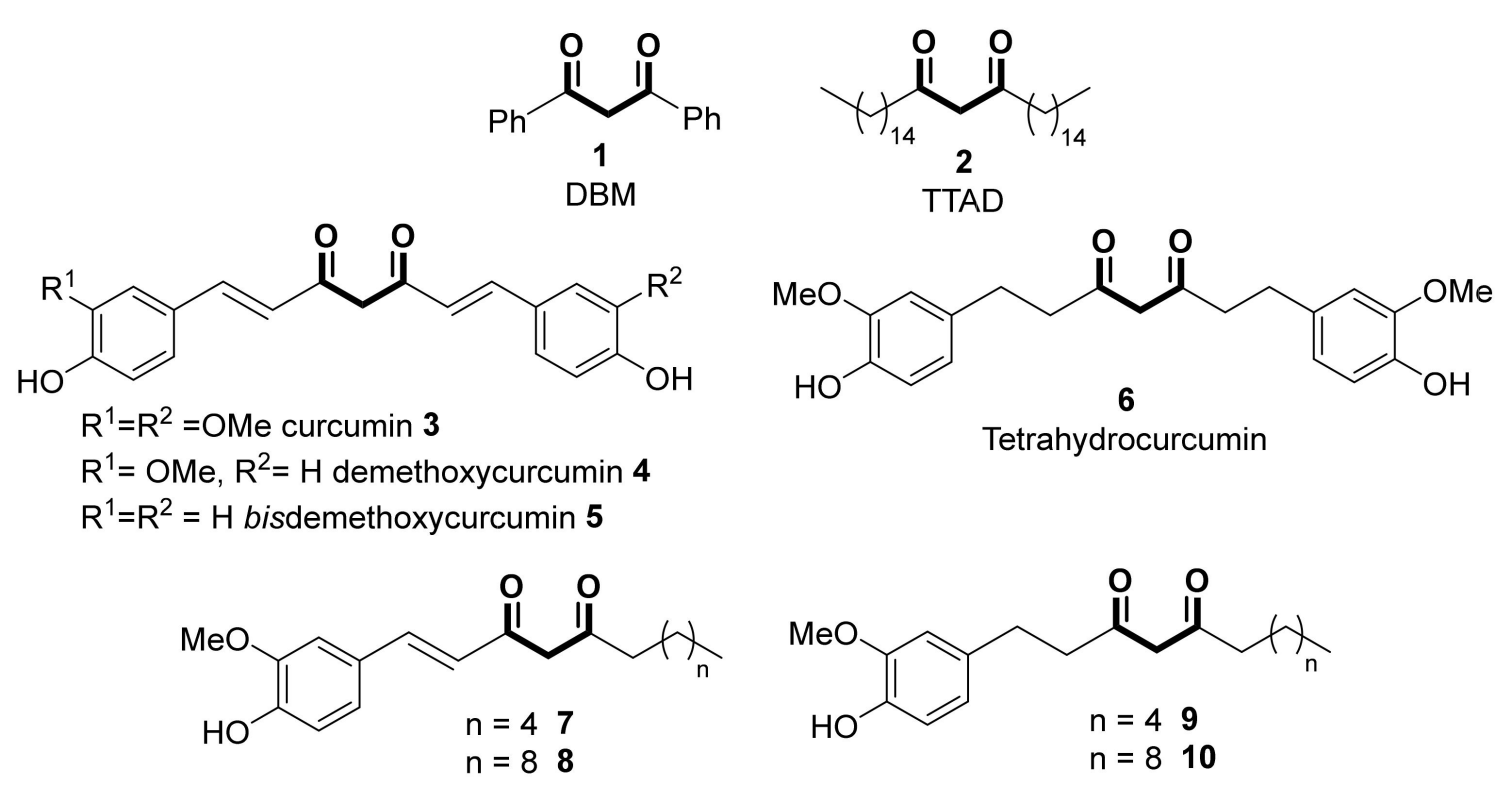
Some linear 1,3-diketones possessing biological activity.

1,3-Diketones (β-diketones) are ubiquitous scaffolds found in many natural products, exhibiting a wide range of biological activities. Thus, many naturally occurring 1,3-diketones shown in Scheme 1, such as dibenzoylmethane (DBM, **1**) or *n*-tritriacontane-16,18-dione (TTAD, **2**), are typical examples of this type of compounds, naturally obtained from plants such as eucalyptus leaves [1,2], licorice roots [3], vanilla beans [4] or sunflower pollen [5]. These powerful natural antioxidants possess prominent anti-cancer properties with minimal toxicity; thus, the potential activity of DBM as a therapeutic option for cancer treatment (in vitro and in vivo activity inhibiting the growth and proliferation of colon, mammary, lung, prostate, neuroblastoma and skin cancers), as well as for diabetes and dementia, has been recently reviewed [6].

Curcumin **3**, an active ingredient of turmeric (a dried, orange-yellow-colored rhizome derived from *Curcuma longa* L.), was for long known to possess antibacterial activity [7]. Curcumin and its mono and bis-demethoxy-derived compounds **4** and **5** are termed curcuminoids, and they account for 2–9% of the active compounds of turmeric [8]. These diarylheptanoids (an aryl-C7-aryl skeleton including the diketone moiety) are considered the principal active constituents responsible for the plethora of biological functions described for turmeric [9]. Among them, curcumin is the most abundant and the most frequently investigated for evaluating its applicability for the prevention and management of many illnesses, including cardiovascular and liver diseases, hypertension, obesity, diabetes, neurological disorders, inflammation, skin diseases, fibrosis, and arthritis [10,11]. The basic mechanism for curcumin’s biological activity is thought to be based on its modulation effect of various signaling molecules, transcription factors, and enzymes [12], as well as regulating epigenetic activity, including the inhibition of DNA methyltransferases (DNMTs), regulation of histone modifications via the regulation of histone acetyltransferases (HATs) and histone deacetylases (HDACs), regulation of microRNAs (miRNA), action as a DNA binding agent and interaction with transcription factors [13]. Additionally, curcumin possesses a very exciting chemo-preventive and therapeutic potential against cancer, as extensively assessed. Tetrahydrocurcumin **6**, the major curcuminoid metabolite of curcumin, displays a higher water solubility compared to curcumin, as well as an enhanced chemical stability, bioavailability, and anti-oxidative activity, so that it could be an excellent alternative [14]. Similarly, the antioxidant activity of components of the ginger (*Zingiber officinale*) rhizome was attributed to [6]– and [10]–dehydrogingerdiones (**7** and **8**) and their corresponding dihydro analogs (**9** and **10**) [15].

**Scheme 2:**
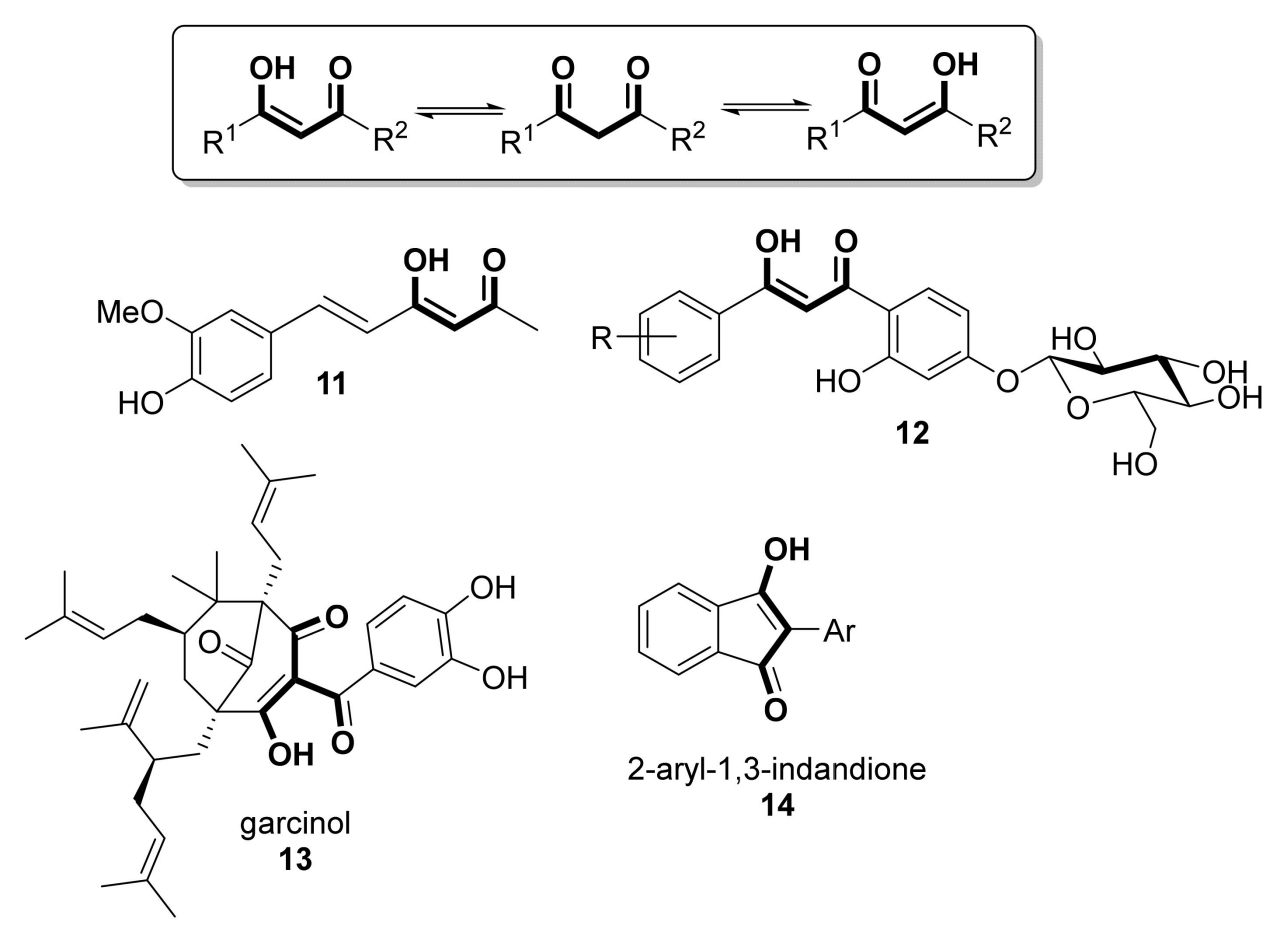
Keto-enol structures of linear and cyclic 1,3-diketones with antioxidant activity.

It has to be taken into account that most of the known dicarbonylic antioxidants exist mainly in the enolized form, as depicted in Scheme 2. In fact, apart from the enolized forms of curcumin, curcuminoids, and phenolic diketones shown in Scheme 1, the antioxidative power of compounds shown in Scheme 2, such as feruloylacetone (**11** [16]), O-β-*D*-glucoside derivatives of β-diketones (**12**, [17]), garcinol (**13** [18], also with potent anti-inflammatory [19] and anti-cancer [20] activities) or 2-aryl-1,3-indandiones (**14**, [21]), has already been reported.

**Scheme 3:**
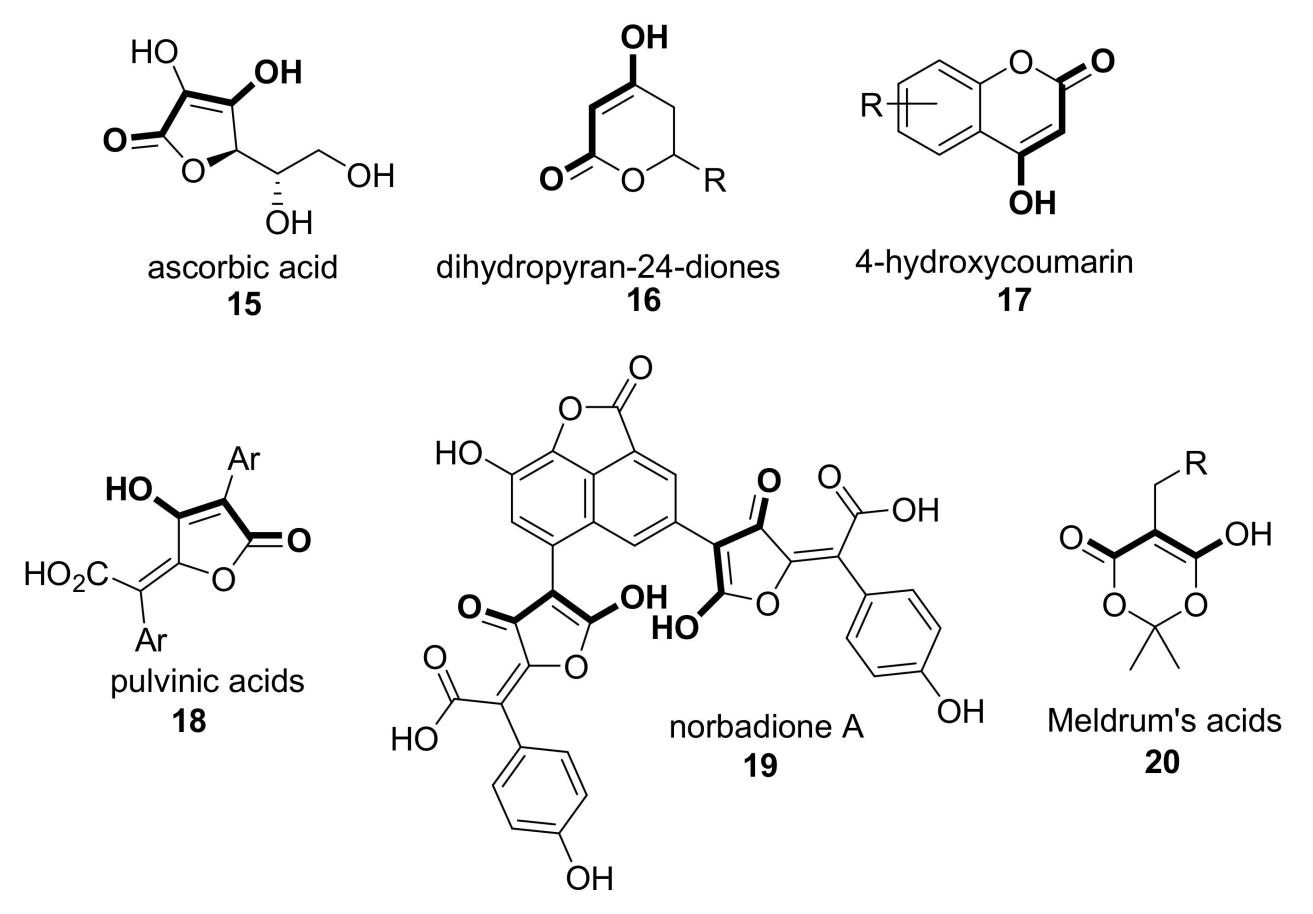
Some examples of β-ketoesters with antioxidant activity.

Similarly, some other antioxidant compounds possessing an enolic moiety can be considered analogs of 1,3-diketones, although they are properly β-ketoesters. Thus, apart from the archetypal ascorbic acid **15** [22,23], compounds such as dihydropyran-2,4-diones **16** [24], 4-hydroxycoumarins **17** [25], pulvinic acids **18** [26,27], and their natural analog norbadione A **19** [28] or Meldrum’s acids **20** have been assessed for their antioxidant capability [29,30], as shown in Scheme 3.

**Scheme 4:**
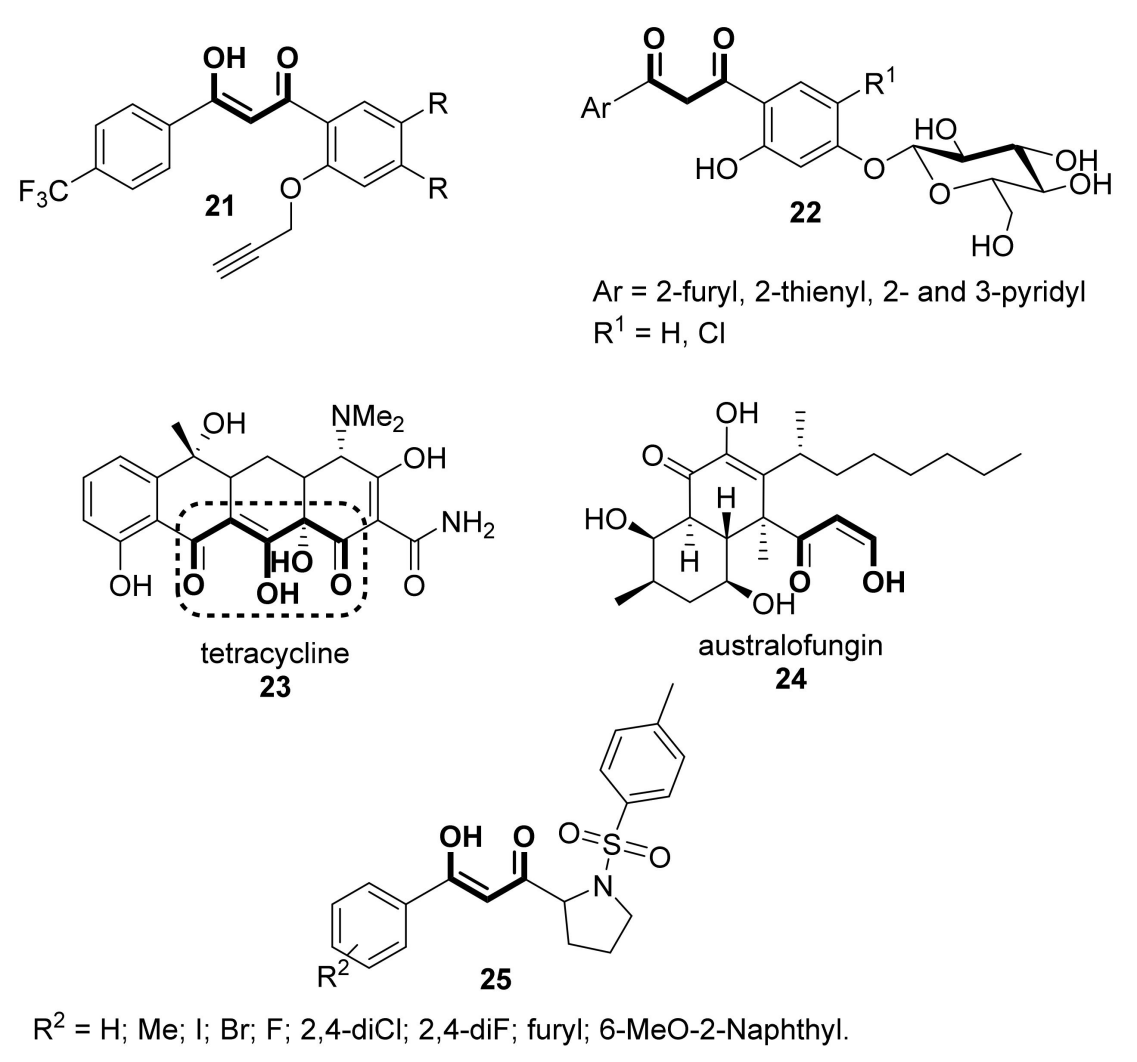
Some linear and cyclic β-diketones possessing different biological activity.

Aside from the antioxidative activity, some other pharmacological activities have been reported for β-diketones or their enolic counterparts. For instance, a series of 3-hydroxy-3-(4′-trifluoromethyl-phenyl)prop-2-en-1-ones (**21**, Scheme 4) has been screened for their anti-malarial activity against chloroquine-sensitive *Plasmodium falciparum* (CD7) [31]. The molecular docking studies with PDB:4ORM confirmed the very potent anti-malarial activity of some of them. On the other hand, water-soluble compounds **22** (Scheme 4), *O*-β-*D*-glucoside derivatives similar to **12** (Scheme 2) have shown antibacterial and antifungal properties [32]. Conversely, also indanones **14** have shown antimicrobial activity [33]. The classical tetracyclines structure **23**, whose use in clinical medicines is well documented [34,35,36,37], contains an α-hydroxy- or an alkoxy- substituent on the central carbon of the 1,3-diketone moiety, as depicted in Scheme 4. These antibiotics, still very useful because of their activity against a broad spectrum of microorganisms and their low toxicity, are being studied as precursors of new drugs [38,39,40]. Australifungin **24**, isolated from *Sporomiella australis,* which contains an unusual β-ketoaldehyde function in its molecular structure, shows a prominent antifungal activity [41]. Additionally, chemical structures possessing the β-diketone structure can be considered as excellent candidates as drugs displaying multi-target potency. For instance, asymmetrical 1,3-diketones **25**, with potential anti-cancer as well as anti-inflammatory activities, were synthesized via direct coupling reaction between ketones and *N*-acyl benzotriazoles, based on soft enolization under mild conditions [42]. These compounds, of which, the drug likeliness and high glycemic index values were also evaluated, were very effective in inhibiting breast cancer cells and the associated inflammation; similarly, these same authors reported some other compounds also possessing an unsymmetrical 1,3-diketone scaffold that is also potentially useful [43].

**Scheme 5:**
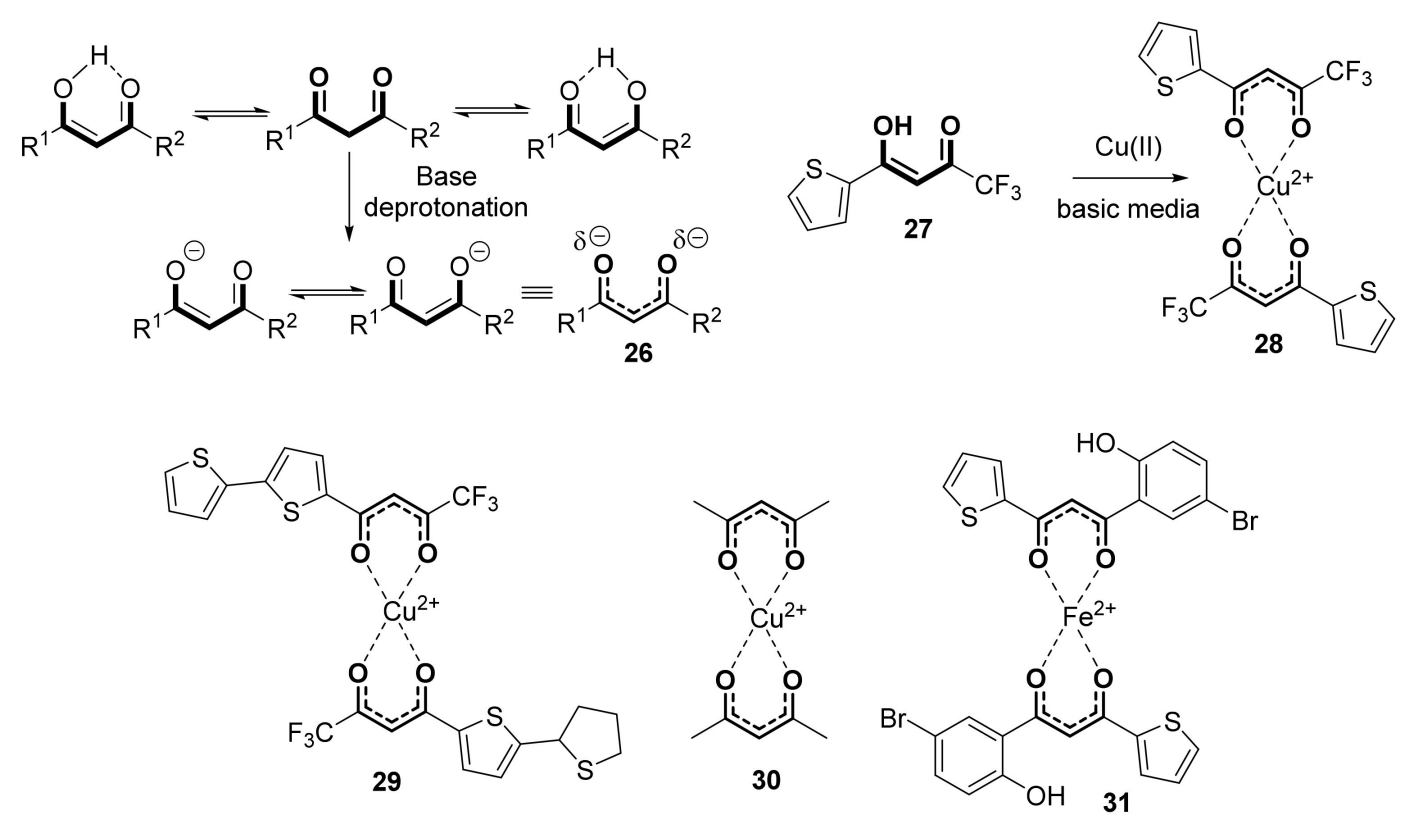
Some 1,3-diketone-metal complexes with antimicrobial activity.

On the other hand, the capability of β-diketones to complex with metals is very well known, through the negative charge of the β-diketonato anion (Scheme 5, **26**), which easily coordinates to many metals such as rhodium [44], manganese [45], iron [46], chromium [47], or copper [48,49]. Thus, they are generally used for solvent extraction of metals from metal-contaminated water [50]. To this respect, a recent theoretical study on the capability of acetyl acetonate anion (Scheme 5, **26**, R^1^ = R^2^ = Me), for complexing Cu(II) has been published [51]. Remarkably, the antimicrobial activity of β-diketones is reinforced when complexed with metals. Thus, coordination of the 2-thenoyltrifluoroacetone **27** (Scheme 5) ligand with copper (II) generated complex **28**, which showed a better antibacterial ability against *Escherichia coli* (ATCC 6358P) and *Staphylococcus aureus* (ATCC 8099) compared to **27** [52]. Similar behavior has been observed for complexes **29** and **30** [53], even showing antifungal activity against *Fusarium verticillioides*. Identical features have been described for Fe (II) complex **31** [54] and some other similar structures [55].

**Scheme 6:**
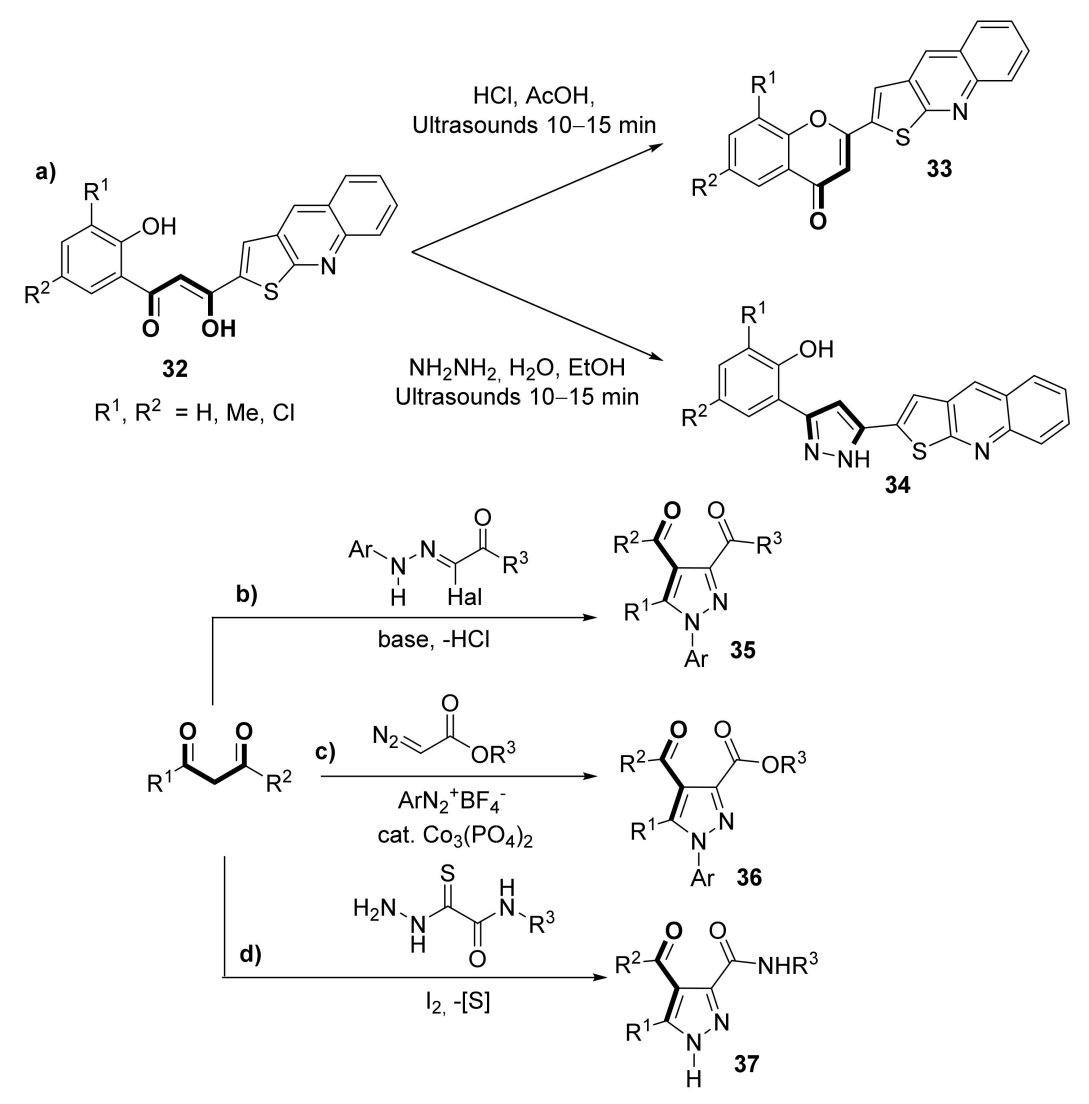
Synthesis of biologically active heterocycles starting from 1,3-dicarbonyl compounds. (**a**) flavonoid type (**33**) and pyrazol type (**34**) compounds with antioxidant and anti-inflammatory activity; (**b**) biologically active *N*-arylpyrazolyl diketones (**35**); (**c**) biologically active *N*-arylpyrazolyl oxoesters (**36**); (**d**) biologically active pyrazolyl oxoamides (**37**).

1,3-diketones can also be used as synthons for furnishing several carbocyclic and heterocyclic structures, as reviewed by Kel’in and Maioli [56], many of them leading to biologically active compounds. Apart from the classical examples covered in that seminal work, many other examples can be found in the literature; specifically, the pyrazol structure is present in many compounds possessing biological activity [57,58,59,60,61], and it is easily accessible through the condensation of 1,3-diketones and hydrazine [57]. In this sense, Mahajan et al. [62] reported the synthesis of thieno [2,3-*b*]quinoline-2 carboxylic acids derivatives **33** (flavonoid type) and **34** (pyrazol type), displaying antioxidant and anti-inflammatory activity, by ultrasonic catalysis starting from asymmetric β-diketone **32** (Scheme 6a). Other commonly used approaches are based on the Michael-type cyclization of 2-halohydrazone acetates with 1,3-dicarbonyl compounds, depicted in Scheme 6b [63,64], or the three-component coupling reaction of aryldiazonium salts with diazo and 1,3-dicarbonyl compounds (Scheme 6c) [65]. Recently, the synthesis of 3,4-dicarbonyl-substituted pyrazoles **37** starting from a wide range of 1,3-dicarbonyl compounds and oxamic acid thiohydrazides was developed via iodine-promoted cascade imination/halogenation/cyclization/ring contraction reaction accompanied by sulfur elimination (Scheme 6d) [66].

## 2. Synthetic Methodologies for the Preparation of β-Diketones

As mentioned in the previous section, β-diketones and their derivatives present high importance as valuable compounds. For this reason, several methodologies have been described for their preparation in different reviews [56,67]. The recent contributions to the preparation of these compounds are discussed in the next paragraphs.

### 2.1. Synthesis of β-Diketones by Claisen Approach

**Scheme 7:**
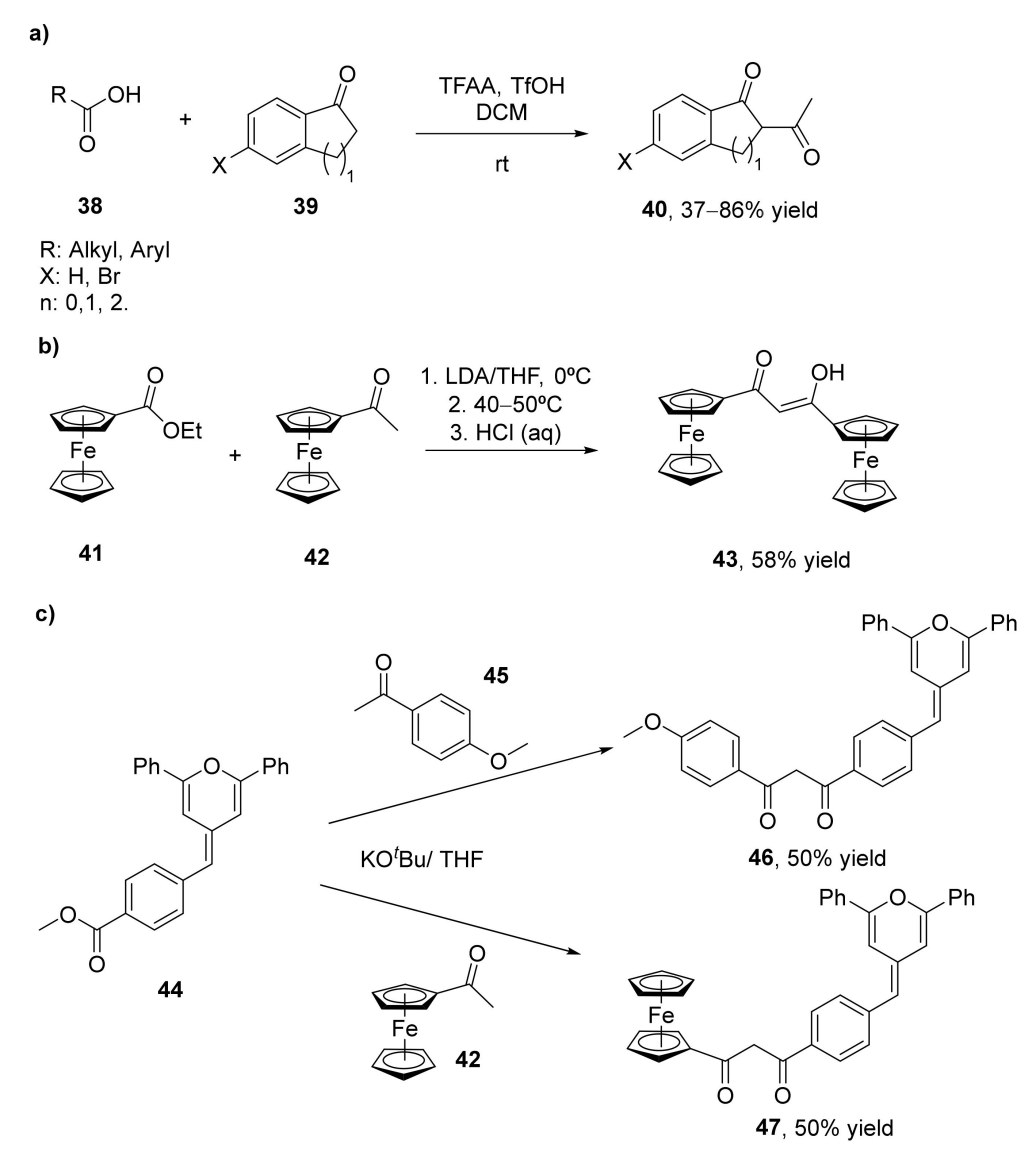
Synthesis of 1,3-diketones through classical Claisen condensation: (**a**) by acylating alkyl aryl ketones (**39**) with alkanoic acids (**38**); (**b**) by the reaction of the ferrocenyl esters (**41**) with different ferrocenyl ketones (**42**); (**c**) by condensation of 4-methylbenzoate methylenpyran (**44**) with different ketones.

The most classical approach for the synthesis of 1,3-diketones consists of the Claisen condensation of ketones and esters under basic conditions. This procedure has been widely explored for several years, and is the most common approach for obtaining these valuable components. Several examples of Claisen condensation improvements have been performed, some of them commented in the present manuscript, for the preparation of 1,3-diketones, but until 2014 no examples of direct synthesis of these compounds from carboxylic acids and ketones had been developed [68]. Initially, it was observed that the treatment of different phenylalkanoic acids (**38**) with trifluoroacetic anhydride (TFAA) and trifluoromethanesulfonic acid (TfOH) yielded the self-acylation compounds (1,3-diketones) as the major products, as a result of the acylation of 1-indanones of 1-tetralones, the initially formed compounds in these processes. This acylation is given mainly when the reactions are carried out in the presence of low amounts of TfOH. When using one equivalent of this acid, the process was completely chemoselective for the formation of the 1,3-diketone, whereas the use of larger amounts resulted in the benzofused ketones as the sole product. In view of these results, the formation of different 1,3-diketones (**40**) was studied by acylating alkyl aryl ketones **39** with alkanoic acids **38**, with yields between 37–86%, as shown in Scheme 7a. Furthermore, the acylation of ketones by carboxylic acids with different functionalities was studied. Thus, the reaction of alanine with 1-indanone, 1-tetralone, and acetophenone led to the corresponding aminodiketones with moderate yields. 

Diferrocenyl β-diketones **43** have also been prepared using a Claisen approach (Scheme 7b) [69]. These compounds have been complexed with different metals and converted into the corresponding pyrazoles, and were characterized and analyzed. The synthesis of these diketones was carried out by the reaction of the ferrocenyl esters **41** with different ferrocenyl ketones **42** in the presence of lithium diisopropylamide (LDA) as a base in tetrahydrofuran (THF) at 0 °C. At these conditions, 54% of the diketone was recovered, but the self-aldol condensation α,β-unsaturated ketone appeared as a byproduct in 17% yield [70]. The use of KO*^t^*Bu as a base reduced this byproduct while slightly increasing the amount of 1,3-diketone (58%).

The Claisen condensation has also been applied for the preparation of anisyl and ferrocenyl adducts of methylenepyran-containing 1,3-diketones, as shown in Scheme 7c [71]. These diketones were obtained from a 4-methylbenzoate methylenpyran (**44**) that was prepared by a Wittig-type reaction between 2,5-dipheny-4*H*-pyran-4-yl triphenylphosphonium tetrafluoroborate salt with methyl 4-formylbenzoate in 75% isolated yield. The Claisen condensation of this benzoate was carried out at room temperature in the presence of KO*^t^*Bu as a base. Thus, a solution of 4-methoxyacetophenone (**45**) or acetylferrocene (**42**) in THF was in situ deprotonated with the base in the presence of two equivalents of **44**. Reaction occurred in 15 h, leading to both β-diketones **46** and **47** in 50% isolated yield. The spectral, structural, and redox properties of both compounds were further analyzed. The authors also described the preparation of extended electron-rich ferrocenylmethylenepyran having a 1,3-diketone coordination site. Thus, ferrocenylmethylenepyran substrate was subjected to Friedel–Crafts acylation in dichloromethane (DCM) at 0 °C for 2 h, leading to the acetylated derivative with 61% isolated yield. This intermediate was treated with lithium diisopropylamide as a base to achieve the ferrocenylmethyelenpyran-containing-1,3-diketone with a 20% yield. 

**Scheme 8:**
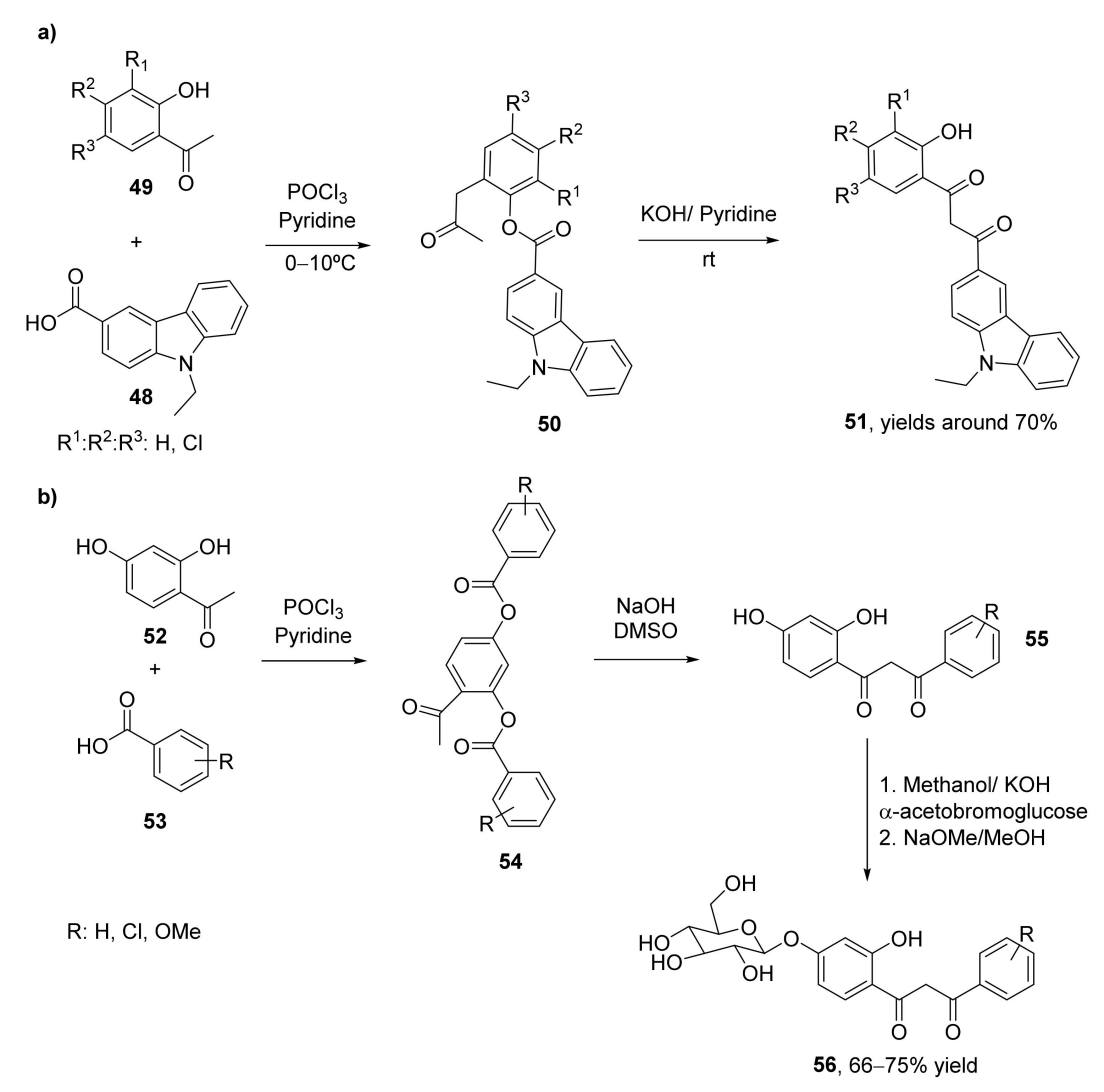
Preparation of valuable β-diketones using the Baker–Venkataraman transposition: (**a**) by treating of carbazole esters (**50**) with KOH in the presence of pyridine; (**b**) by treating diester **54** with NaOH/DMSO.

Carbazole derivatives present a wide set of biological activities as antimicrobial [72], antiviral [73], antitumoral [74], or anti-inflammatory [75] compounds. For this reason, a set of carbazole-based 1,3-diketones (**51**) have been recently synthesized and evaluated as antimicrobial molecules [76]. 9-Ethyl-9*H*-carbazole-3-carbaldehyde was oxidized to 9-ethyl-9*H*-carbazole-3-carboxylic acid **48**, which was converted into the corresponding esters by treatment with 2-hydroxyacetophenones (**49**) in the presence of phosphorous oxychloride and pyridine. These carbazole esters (**50**) were treated with KOH in the presence of pyridine, leading to an intramolecular Claisen condensation through a Baker–Venkataraman transposition, leading to the corresponding 1,3-diketones with yields of around 70% (Scheme 8a). These diketones were further transformed into the carbazole pyrazoles, whose biological properties were also evaluated. 

The Baker–Venkataraman transposition has also been employed for the synthesis of a set of *O*-β-*D*-glucoside derivatives of β-diketones (**56**, Scheme 8b) [17], whose antibacterial, antifungal, and antioxidants activities have been evaluated. 2,4-Diaryloxyacetophenones **52** and benzoic acids **53** were subjected to the transposition employing phosphorous oxychloride and pyridine in a first step to obtain compounds **54**, which lead to the 1-(2′,4′-dihydroxyphenyl)-3-aryl-propane-1,3-diones (**55**) with yields between 64% and 74% after treatment with NaOH in dimethylsulfoxide (DMSO). The corresponding β-diketones were dissolved in 2.5% methanolic KOH and coupled with α-acetobromoglucose under nitrogen atmosphere to obtain the corresponding glycosylated 1,3-diketones with yields of around 70%. Finally, the tetra-*O*-acetyl-*O*-β-*D*-glucosides were deacetylated in dry methanol with sodium methoxide under an inert atmosphere, leading to the 1-(4′-*O*-β-*D*-glucopyranosyloxy-2′-hydroxyphenyl)-3-aryl-propane-1,3-diones also with isolated yields between 66–75%.

#### 2.1.1. Preparation of Halogenated Diketones

**Scheme 9:**
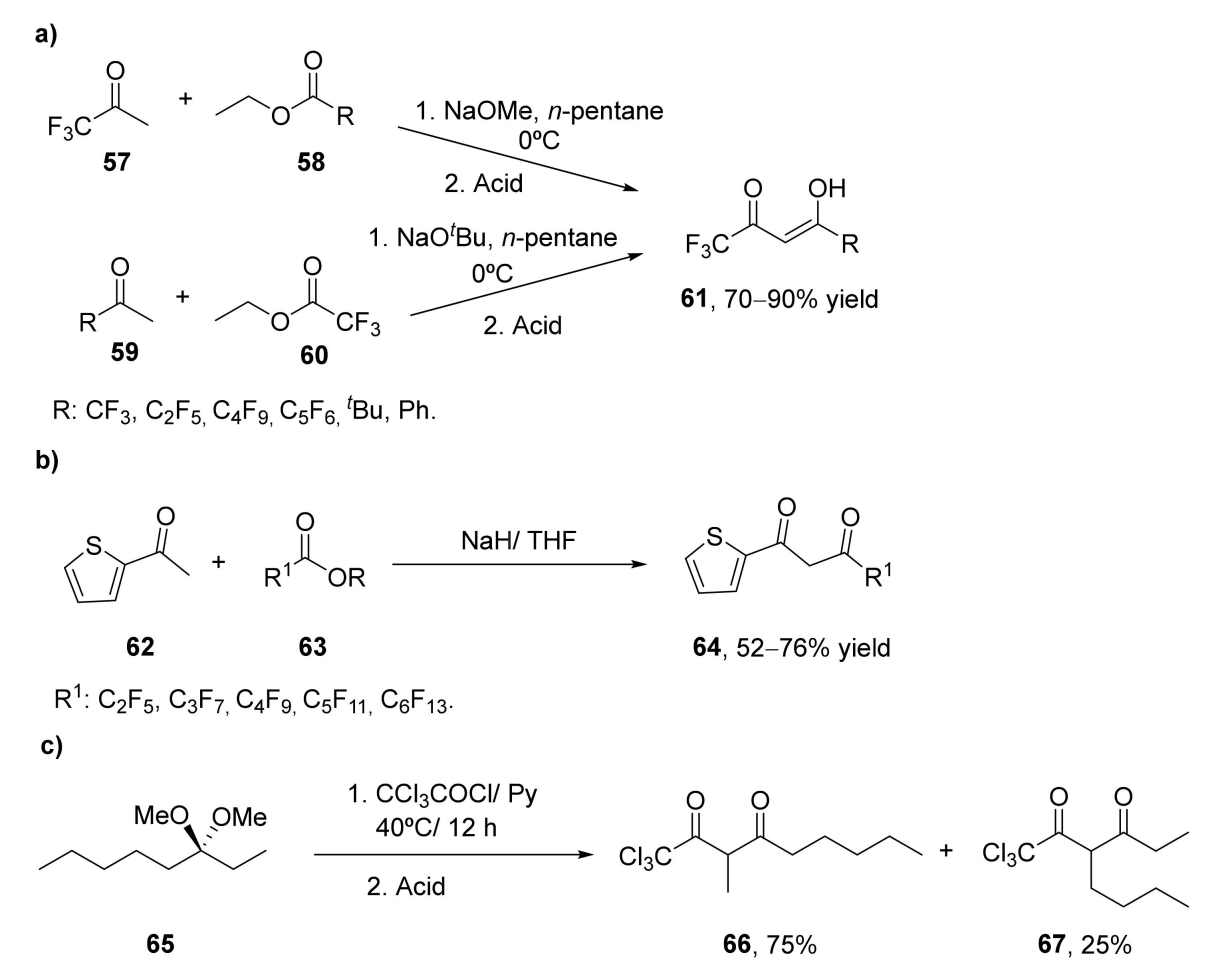
Preparation of halogenated 1,3-diketones via Claisen condensation: (**a**) via condensation of fluoroketones (**57** or **59**) with perfluoro alkylesters (**58** or **60**); (**b**) by condensation between 2-acetylthiophene (**62**) and fluorinated esters (**63**); (**c**) by acetal acylation with trichloroacetyl chloride.

Besides the importance of classical β-diketones, those bearing (per)halogenated groups are of high interest due to their applications in different fields. In addition, the incorporation of these halogen atoms into the organic molecules often led to improved stability and pharmacokinetic properties [77,78]. In the last few years, different approaches have been developed in order to obtain these valuable materials. Thus, in 2016, the synthesis of a set of pefluroroalkyl substituted β-diketones **61** (Scheme 9a) were prepared by an improved Claisen-condensation of 1,1,1,-trifluoroacetone **57** and the corresponding perfluoroalkylesters **58**, or well by the condensation of halogenated acetones **59** and ethyl 2,2,2-trifluoroacetate **60** in the presence of different alkoxides using *n*-pentane as solvent [79]. The so-obtained diketones **61** were purified by forming copper complexes insoluble in water that can be separated from the starting materials. In general, good to excellent yields were achieved after the purification step. These compounds were further employed for the synthesis of trifluoromethyl pyrazoles, compounds that present biological activity, as commented before.

In 2018, a method for the preparation of a set of 2-thienyl diketones **64** with different lengths of a perfluorinated side has been described, as shown in Scheme 9b [80]. Initial studies were carried out by the Claisen condensation between 2-acetylthiophene **62** and fluorinated esters **63** in the presence of different bases employing anhydrous Et_2_O as a solvent in the presence of highly active alkoxides as NaOMe or NaOEt. The higher reaction yields were achieved when a mixture of both the ester and the ketone was added to a suspension of the base, whose quality presented a significant effect on the reaction yield. As the degradation of sodium alkoxides was difficult to control under the laboratory conditions, authors have analyzed NaH as an alternative base, optimizing the rest of the reaction conditions. Thus, THF was a more suitable solvent for this process, finding an optimal molar ratio between ester, ketone, and base of 1:1:2. The reaction was performed at 5 °C, and once completed, it was stored at room temperature for 5–10 h, achieving the 1,3-diketone with 92% total yield when using methyl heptafluorobutanoate as test substrate. This procedure was extended to the preparation of other perfluorinated 1,3-diketones, obtaining in all cases good yields. 1-(2-Thienyl)butane-1,3-dione was prepared in 71% yield through the condensation of 2-acetylthiophene with ethyl acetate. Further purification of the 1,3-diketones obtained by Claisen condensation was performed by forming the copper salts of the diketone by treatment with Cu(OAc)_2_ in the presence of acetic acid and water. The decomposition of the copper chelate was carried out by the addition of Na_2_EDTA in a biphasic system water/EtOAc. 

The synthesis of 1,1,1-trichloro-4-methoxy-3-alken-2-ones and 1,1,1-trichloro-2,4-alkadiones presenting long alkyl chains with high yields has been recently reported from acetal acylation with trichloroacetyl chloride [81]. The dimethoxy acetals were prepared by acetalization of the fatty ketones with trimethylortoformate and *p*-toluensulfonic acid. These acetals were then subjected to trichloroacetylation in chloroform and pyridine, achieving the alken-2-ones as final products with high yields (85–95%); however, at the reaction conditions, with work-up in the presence of HCl and water, octan-3-one (**65**) is converted in the trichloromethyl-β-diketones, thus achieving the mixture of 1,3-diketones 1,1,1-trichloro-3-methylnonanon-2,4-dione (**66**) and 1,1,1-trichloromethyl-3-hexan-2,4-dione (**67**) in a 75:25 proportion, respectively, as shown in Scheme 9c. 

#### 2.1.2. Strategies for the Preparation of 1,3-Diketones Using Other Enolates

**Scheme 10:**
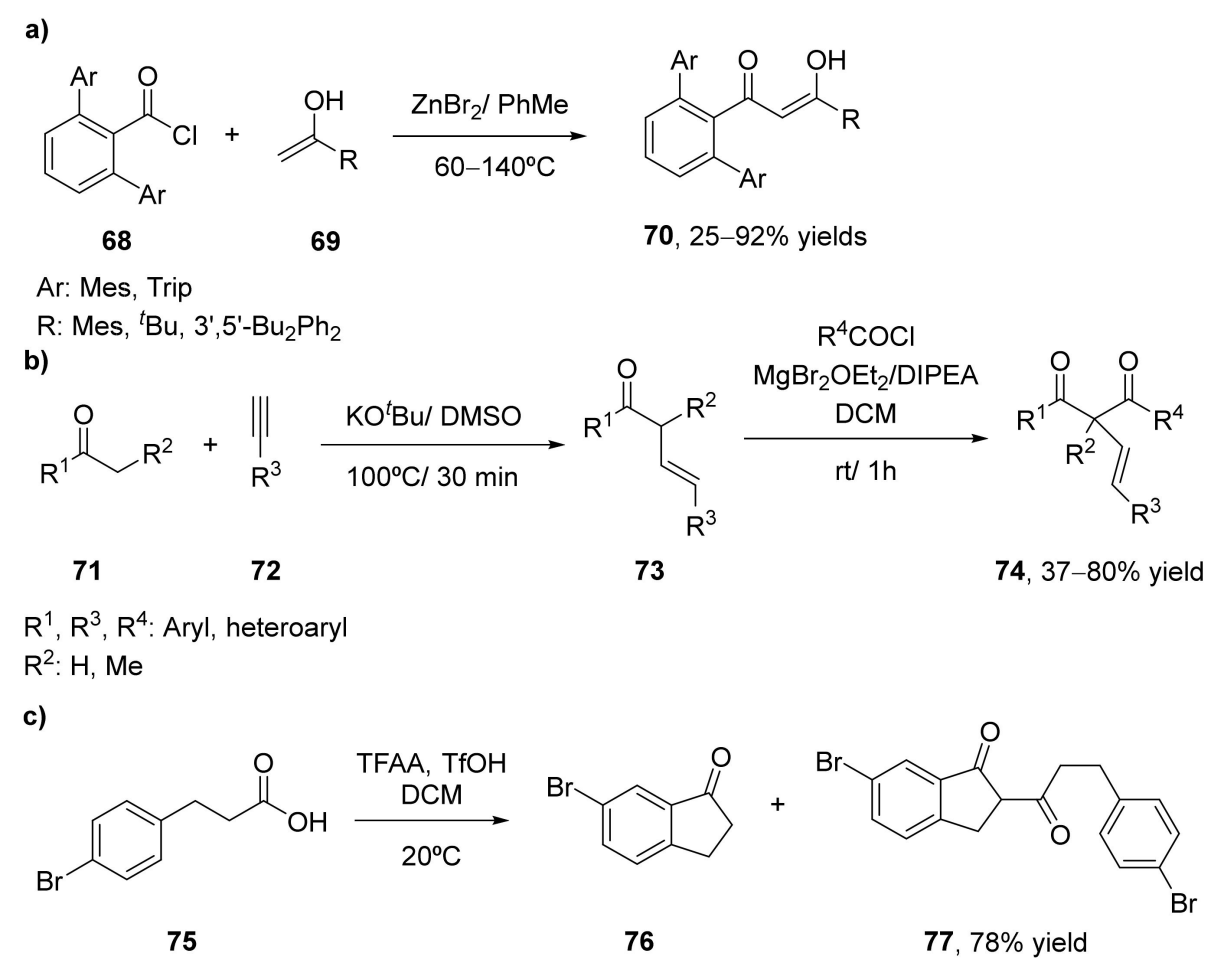
Preparation of 1,3-diketones using soft enolates methodologies: (**a**) by using acid chlorides (**68**) as electrophiles; (**b**) by using acid chlorides and β,γ-unsaturated ketones (**73**); (**c**) starting from β–phenylpropionic acids (**75**).

The classical Claisen reaction typically requires a strong base. These reaction conditions are a limitation for the preparation of β-diketones containing base-sensitive substituents. For this reason, soft enolization techniques have been developed, in order to increase the efficiency of the coupling. One example was shown in 2019, employing acid chlorides as electrophiles in the condensation process for the preparation of a set of sterically hindered 1,3-diketones **70** containing the dipivaloyl methane group [82], as shown in Scheme 10a. Initial studies were performed in the reaction of the electrophiles pivaloyl chloride or methyl pivalate (**68**) with sodium pinacolonate (**69**) to yield the corresponding 1,3-diketone. Different solvents were tested, achieving similar yields (around 65% after 2 h at room temperature) when using pivaloyl chloride in the presence of toluene, DCM, or THF, although toluene was more effective if the reaction was left overnight. Reactions with methyl pivalate led to much lower yields even when heating. The process in the presence of the acid chloride was scaled up to a multigram scale, affording the dipivaloyl 1,3-diketone with 88% yield. The process was also studied employing another bulky electrophile as 2,6-dimesitylbenzoyl chloride with the sodium enolate from trimethylacetophenone. The reaction was performed in the presence of a Lewis acid, to diminish a possible *O*-acylation product, resulting ZnBr_2_ as the best compound for obtaining the 1,3-diketone, with the complete conversion after 2 days at 60 °C in toluene. Only traces of the *O*-acylation compound were achieved under these conditions. This process was extended to other bulky enolates in order to prepare the bulky 1,3-diketones, and it was observed that when the use of ZnBr_2_ is sensitive to the solvent employed, as a significant amount of *O*-acylation can be observed, whereas employing NaBr, no effect of the solvent nature was observed, achieving in general, good yields. The reaction between 2,6-dimesitylbenzoyl chloride with the sodium enolate from *tert*-butyl methyl ketone was scaled up to 10 g.

In 2020, a novel method for the preparation of α-alkenyl-β-diketones **69** had been developed starting from β,γ-unsaturated ketones in the presence of acid chlorides [83], as depicted in Scheme 10b. The β,γ-unsaturated ketones were prepared by the functionalization of the ketones **71** with acetylenes **72** at 100 °C in the presence of KO*^t^*Bu/DMSO as a catalytic system. In view of that, the classical Claisen conditions failed to prepare the desired 1,3-diketones, a soft enolization procedure was carried out. Thus, a coordination complex between a Lewis acid as magnesium bromide and the β,γ-unsaturated ketone **73** provide the enolate. The presence of the Lewis acid increases the polarization of the carbonyl group, increases the acidity of the α-proton and blocks the oxygen-atom in order to prevent *O*-acetylations. The corresponding α-alkenyl-β-diketones (**74**) were prepared by adding magnesium bromide etherate and the β,γ-unsaturated ketone into a solution of the corresponding acyl chloride in DCM. The reaction was stirred for 5 min at room temperature, and then *N*,*N*-disopropylethylamine was added, reacting at room temperature. When using benzoyl chloride, the final 1,3-diketone was obtained in 72% yield after 1 h. Lowering both the Lewis acid or the base led to a decrease in the yields, whereas the reaction in the absence of magnesium salt only afforded the *O*-acetylation product. The acylation of the unsaturated ketone was tested in the presence of other aryl and heteroaryl chlorides, leading to the final 1,3-diketones in good yields (48–80%). Only when employing isobutyryl chloride, the diketone was not achieved, where we observed only the *O*-acetylation product. The use of other β,γ-unsaturated ketones having a hydrogen substituent instead of a methyl group at the α position was more complicated, due to the instability of the final 1,3-diketones when subjected to purification by column chromatography, as partial isomerization and decomposition were observed. 

An aromatic 1,3-diketone bearing a thiophene moiety has been recently described, also employing soft enolization techniques [84]. This compound has further been employed as a ligand in iridium complexes with interesting photochemical properties. The synthesis was carried out in a two-step process. Initially, a benzotriazole amide of isophthalic acid monomethyl ester was prepared, which was employed as the soft acylation reagent with 2-acetylthiophene in DCM at 20 °C using MgBr_2_·Et_2_O as Lewis acid and with *N*,*N*-disopropylethylamine as a base. The desired 1,3-diketone was recovered with 58% yield after 20 h reaction.

β–Phenylpropionic acids have been employed as starting materials in the formation of 1,3-diketones through intra- and intermolecular self-acylation reactions in the presence of the system TFAA-TfOH [85], as shown in Scheme 10c. TFAA was employed as a reaction medium and as an activator to convert carboxylic acids into mixed anhydrides, acyl trifluoroacetates, which are efficient acylating agents, while TfOH helps to the enolization and increases the acylating power of acyl trifluoroacetates. β–Phenylpropionic acid was dissolved in a mixture of TFAA and DCM in the presence of 0.25 equivalents of TfOH at room temperature, yielding a 55% of 2-(3-phenyl-1-oxopropyl)indan-1-one and only 2% of 1-indanone, the product of the acid-catalyzed intramolecular cyclization of the carboxylic acid, intermediate that was then acylated to yield the desired 1,3-diketone. If the amount of TfOH is increased to 0.5 equivalents, an 80% of the desired product was recovered. Opposite, the reaction of γ-phenylbutyric acid at the same conditions only afforded the intermediate tetralone, with no further acylation process. When starting form 3-(4-bromophenyl)propionic acid **75**, as depicted in Scheme 8c, 67% yield of the diketone **77** was obtained with 0.5 equivalents of TfOH. Increasing up to 1.0 equivalent led to a 78% yield, whereas higher amounts of the acid (3.0 equivalents) only afforded 6-bromo-1-indanone **76** as a final product. Finally, 3-[4-(1-adamantyl)phenyl)]propionic acid was treated at the same conditions (0.5 equivalents of TfOH) to obtain a 35% of **77** and 48% of **76**, which was not able to further react at the reaction medium due to its low selectivity. In further development, the obtained 1,3-diketones were converted into the corresponding pyrazoles, compounds of high interest with valuable biological properties, as previously commented.

### 2.2. Synthesis of 1,3-Diketones by Hydration of Alkynones

Alkynones are valuable structural motifs in organic chemistry, normally prepared by the Sonogashira coupling of terminal alkynes with different acyl chlorides. The hydration of these compounds leads to the formation of 1,3-diketones, but the requirement of strong acidic conditions or the presence of PtCl_4_ or amines as catalysts reduces the application of this synthetic methodology. For this reason, in the last years, some other approaches for the hydration of alkynones under mild reaction conditions have been described [86,87].

**Scheme 11:**
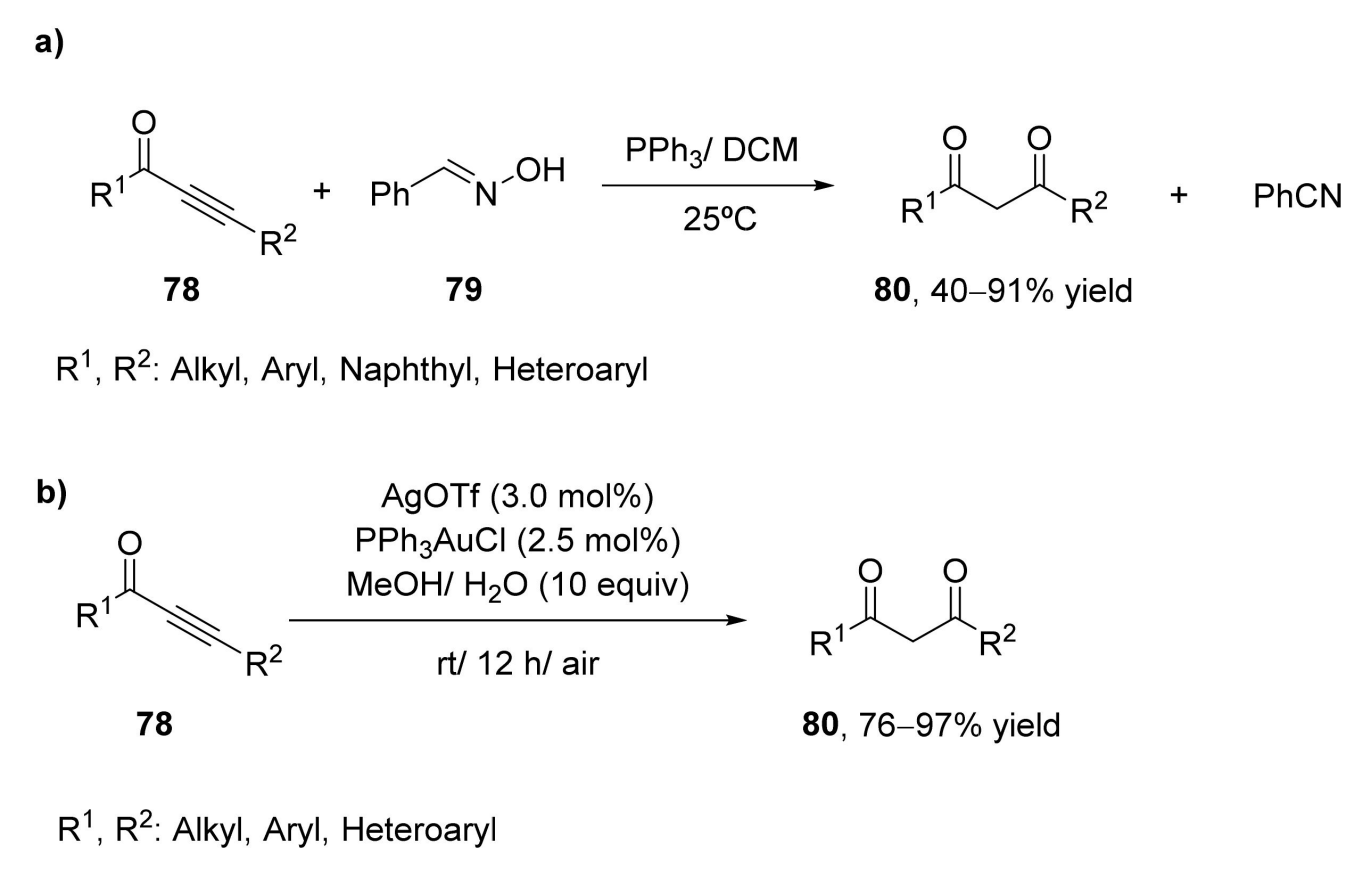
Preparation of 1,3-diketones by hydration of alkynones (**78**): (**a**) by treating with benzaldehyde oxime (**79**); (**b**) by gold(I)-catalyzed regioselective hydration.

Thus, in 2018, the application of oximes for the formation of 1,3-diketones from the corresponding alkynones was carried out [88]. 1,3-Diphenylprop-2-yn-1-one (**78**, R^1^ = R^2^ = Ph) was treated with benzaldehyde oxime **79** for the synthesis of the corresponding 1,3-diketone, as shown in Scheme 11a. After analyzing different reaction conditions, the optimized process was performed using dichloromethane as solvent and 1.1 equivalents of oxime and 20 mol% of PPh_3_ as a catalyst. At room temperature, 87% of the diketone was recovered after 3 h. The reaction was extended to other alkynones presenting alkyl, halo, and cyano substituents at the *orto-*, *meta-,* and *para*-positions of the aromatic ring with high yields. This method was also useful for naphthyl and heteroaryl alkynones, but no reaction was observed when the carbonyl moiety was linked to an aliphatic carbon atom. The use of aromatic and aliphatic aldehyde oximes resulted in the formation of the 1,3-diketone s with good to high yields.

A set of aromatic and heteroaromatic 1,3-diketones **80** have been recently prepared by a gold(I)-catalyzed regioselective hydration of alkynones at mild reaction conditions [89]. Substrate presenting R^1^ = Ph; R^2^ = *n*-Bu was employed as a model substrate in the presence of PPh_3_AuCl (5.0 mol%) as a catalyst in methanol containing AgOTf (6 mol%) and five equivalents of water. Under these conditions, the regioselective hydration of the alkyne was performed successfully to obtain the desired product with 98% yield after 12 h. These optimized conditions were extended to other alkynones. The hydration of different ketones containing an *n*-butyl group at the alkyne terminal afforded the 1,3-diketones with excellent yields, even when employing aryl, heteroaryl, or alkyl groups at the carbonyl side of the starting material. The effect of the substituents at the alkyne terminal was also analyzed at the optimized conditions. Ketones bearing a phenyl group at the terminal carbonyl were tested, we observed that both aromatic and aliphatic substituents were good substrates for this procedure, achieving yields between 80–96%. 

This methodology was carried out at gram-scale for some of the starting ketones, achieving the final 1,3-diketones with yields around 90%, even when using lower catalysts loading (1.0 mol% of PPh_3_AuCl and 1.2 mol% of AgOTf).

### 2.3. Decarboxylative Coupling Reactions for the Synthesis of 1,3-Diketones 

**Scheme 12:**
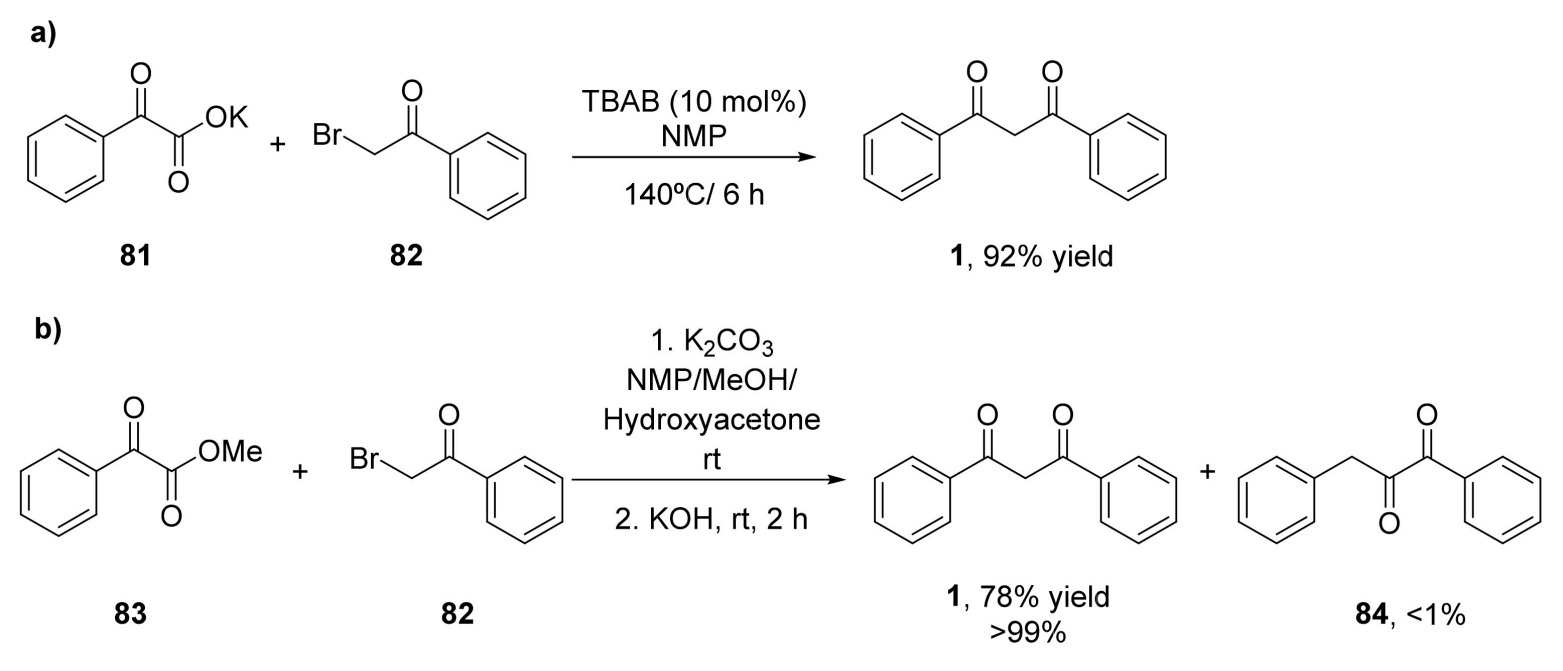
Preparation of β-diketones by decarboxylative coupling reactions: (**a**) cross-coupling between potassium 2-oxo-phenylacetate (**81**) and 2-bromo-1-phenylethanone (**82**); (**b**) decarboxylate coupling between **82** and methyl 2-oxo-2-phenylacetate (**83**).

The decarboxylative coupling reactions catalyzed by transition metals have been established as a useful methodology for the formation of C–C bonds [90]. α-Oxocarboxylic acids have been employed as valuable acyl anion reagents, generated in situ by extrusion of carbon dioxide in the presence of the transition metals [91]. These reagents have been used for the acylation of arenes, enamides, and formamides, but no examples of sp^3^-hybridized carbon centers have been reported until 2015, in which a set of α-bromoketones reacted with α-oxocarboxylates to yield the corresponding β-diketones in the absence of transition metals [92]. Initial experiments analyzed the cross-coupling between potassium 2-oxo-phenylacetate (**81**) and 2-bromo-1-phenylethanone (**82**) in the presence of different catalysts at 140 °C to yield 1,3-diphenylpropane-1,3-dione (**1**, Scheme 12a). It was observed that the control reaction in the absence of catalyst afforded higher yields. Mechanistical studies showed that KBr, that is the second product formed in the metathesis of the carboxylate and the bromoketone, was the catalysts in the decarboxylation to yield the 1,3-diketone. Thus, the reaction performed in the presence of KBr afforded the desired product in 63% yield, whereas other potassium halides led to lower yields. The best result was obtained in the presence of tetrabutylammonium bromide (TBAB, 86% yield), as the cation of this salt serves as a phase transfer catalyst. *N*-Methyl-2-pyrrolidone (NMP) was the best solvent for the reaction, which can be further optimized by increasing the temperature to 160 °C, achieving the diketone **1** with 92% yield. Once optimized the process, a set of potassium α-carboxylates were tested in this reaction. Final 1,3-diketones were obtained with yields from 52% to 81%. Those aryl α-carboxylates containing electron-withdrawing groups led to the diketones with good yields, whereas worse results were obtained with electron-donating compounds. Heteroaryl diketones were recovered with moderate yields. Regarding the α-bromoketones, both aromatic and aliphatic substituents can be employed, achieving in general moderate to high yields, demonstrating that this procedure was a versatile method for the preparation of a wide set of β-diketones.

In 2017, the decarboxylate coupling between methyl 2-oxo-2-phenylacetate (**83**) and **82** in the presence of K_2_CO_3_ using NMP as solvent at room temperature was performed. After 24 h, hydrolysis in the presence of KOH was carried out for 2 h, and a mixture 11:9 of 1,2-diketone **84** and 1,3-diketone **1** with a 36% yield was achieved (Scheme 12b) [93]. Different solvents and bases were studied in this process, and it was discovered that when the process was performed in a mixture of NMP, methanol, and hydroxyacetone, with a further treatment with 2.0 N KOH, a 78% of the 1,3-diketone **1** (>99:1 ratio with the 1,2-diketone) was recovered. These optimal conditions for the preparation of the 1,3-regioisomers were extended to other α-bromoketones and α-oxocarboxylates, the corresponding 1,3-diketones with moderate to good yields were obtained. The steric effect on the α-oxocarboxylate has a greater influence on the reaction yield than the one from the α-bromoketone. Those substrates containing heteroaryl substituents were also accepted and even an α,β-unsaturated α-bromoketone was employed, and the 1,3-diketone with 63% yield was recovered. Finally, an aliphatic oxocarboxylate (cyclohexyl derivative) led to the final compound with a 67% yield. 

### 2.4. Miscellaneous Methodologies 

**Scheme 13:**
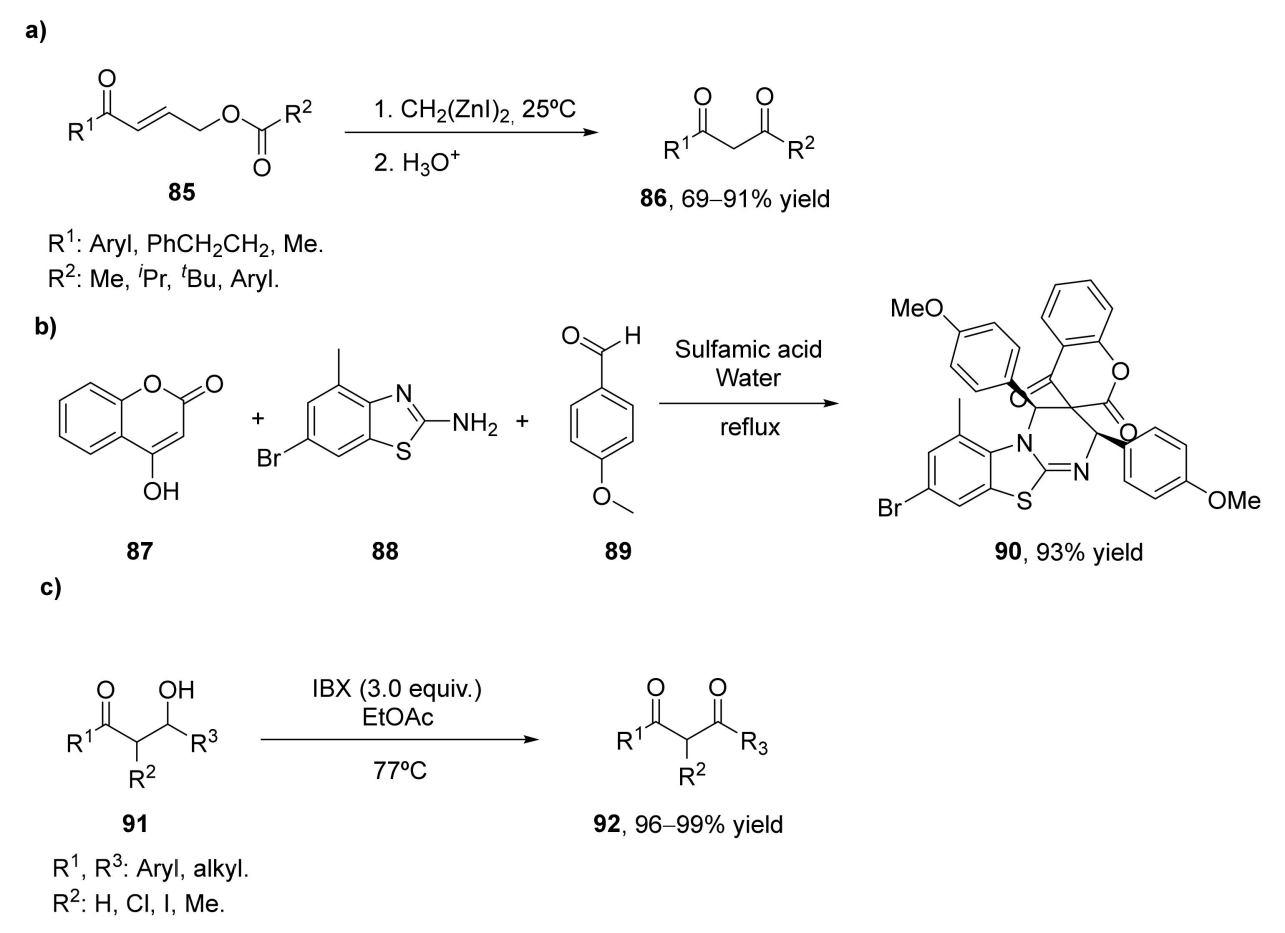
Different synthetic methodologies for the preparation of β-diketones: (**a**) 1,4-addition of bis(iodozincio)methane onto α.β-unsaturated ketones **85**; (**b**) via a multicomponent process; (**c**) by oxidation of β-hydroxyketones (**91**) with o-iodoxybenzoic acid (IBX).

Bis(iodozincio)methane has been employed in the 1,4-addition onto α.β-unsaturated ketones **85**. The use of this reactive in stoichiometric amounts in the presence of chlorotrimethylsilane afforded (*Z*)-silyl enol ethers of β-zinciomethylated ketones. The silylation reagent can be eliminated by using an acyloxy group at the γ-position of the unsaturated ketone, it is possible in this way to convert (*E*)-4-acetoxy-1-phenyl-2-buten-1-one into the desired 1,3-diketone with 91% yield while working in THF at room temperature for 2 h (Scheme 13a) [94]. This methodology was extended to the preparation of other 1,3-diketones (**86**). Aryl enones led to the final products with excellent yields (84–99%), whereas those alkylic ones afforded the diketones with moderate yields (51–69%). As the starting enones can be easily prepared by esterification of the corresponding alcohols, the tandem process represents a valuable method for the preparation of 1.3-diketones. 

Mechanistical studies on this process showed that there is an elimination of three atoms in the last step of the tandem reaction. Thus, when this process was also applied to a lactone as starting material, a 1,3-diketone with a three-atom contraction in the lactone ring was achieved as the final product. 

Spiroheterocyles present high importance in organic synthesis as well as in medicinal chemistry. The rigidity of these spirocycles led to novel biological and pharmaceutical properties for this reason. Most of the syntheses of these spiroheterocycles have been carried out using multicomponent reactions using normally non-environmentally friendly reagents. In 2017, a multicomponent process for the preparation of some of these compounds containing 1,3-diketones was developed [95], as shown in Scheme 13b. Thus, the reaction of 1 equivalent of 4-hydroxycoumarin (**87**) and 2-amino-6-bromo-4-mehtylbenzothiazole (**88**) with two equivalents of *p*-anisaldehyde (**89**) was studied at different conditions. The reaction had a better yield in protic solvents such as methanol, ethanol, and water, whereas the amount of the reaction catalyst (sulfamic acid) was optimized, it was observed that with 2 mol% is more than enough to ensure a proper reaction. Thus, the best conditions were observed with sulfamic acid-water at reflux, achieving a 93% yield after 10 min. The multicomponent process proceeds with the condensation of the hydroxycoumarin with the aldehyde forming the first intermediate. The second equivalent of the aldehyde reacts with the thiazole in the presence of the acid catalyst to form an imine. The hetero-Diels–Alder reaction between the first intermediate and the imine led to the spiro[pyrimido[2,1-*b*]benzothiazole-3,3′-chromene]-2′,4′-dione **90**.

The oxidation of β-hydroxyketones (**91**) by different oxidants can also be a good approach for the preparation of β-diketones (**92**), but only a few examples have been described in the bibliography. In 2011, it was shown the preparation of these valuable compounds with high yields employing *o*-iodoxybenzoic acid (IBX), as shown in Scheme 13c [96]. Initial studies showed that this oxidant led to much better results in the oxidation than the application of the Swern oxidation or the use of Dess–Martin periodinane. IBX was then tested in the oxidation of different aldol-type substrates, achieving the corresponding 1,3-diketones in nearly quantitative yield. Both alkyl and aryl substituents are tolerated, as well as acyclic and cyclic β-hydroxyketones. *Syn*- and *anti*-aldol diastereomers are also oxidized with excellent yields. An additional advantage of this method is that the reaction work-up is very simple, as the reaction mixture is filtered through silica gel to remove the heterogeneous oxidant.

## 3. Catalytic Methods for the Preparation of 1,3-Diketones 

Catalytic approaches have also been employed for obtaining 1,3-diketones. Depending on the nature of the catalyst, these compounds have been synthesized by using enzymes, organocatalysts, or metal-based complexes; in the following, we describe some examples in the last few years. 

### 3.1. Biocatalytic Synthesis of 1,3-Diketones

**Scheme 14:**
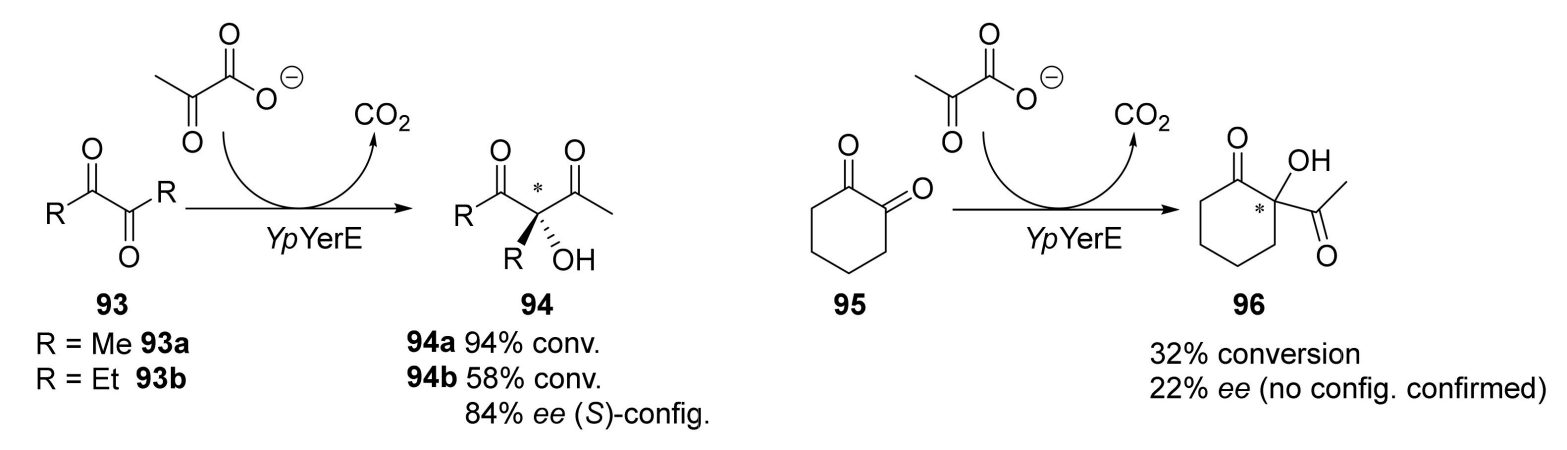
Preparation of a set of 1,3-diketones by a biocatalytic approach employing *Yp*YerE.

Biocatalysts has proven to be an excellent and greener complement for classical organic synthesis, as the employ of biocatalysts (either native, chemically, or genetically modified) allows very clean and selective procedures [97,98,99,100,101,102,103,104,105,106]. Regarding the biocatalytic synthesis of 1,3-diketones, a straightforward methodology was initially reported in 2010 by Müller and coworkers [107] by using a recombinant enzyme (YpYerE, a thiamine diphosphate (ThDP)-dependent aldolase from *Yersinia pseudotuberculosis* O:VI). This enzyme is capable of catalyzing the cross-aldol condensation between aldehyde (obtained via in situ decarboxylations of pyruvate) and ketones, leading to chiral α-acyl tertiary alcohols. The biocatalyst exhibits a very broad substrate tolerance, accepting cyclic and open-chain ketones, as well as diketones and α- and β-ketoesters as acceptor substrates. Thus, when this accepter is an α-diketone, the final product is a β-diketone with two substituents (alkyl and hydroxyl) in the central methylene, as shown in Scheme 14.

This same research group reported the use of another recombinant enzyme, the double variant CDH-H28A/N484A obtained from the ThDP-dependent cyclohexane-1,2-dione hydrolase (CDH) from *Azoarcus* sp. strain 22Lin expressed in *E. coli* [108]. This enzyme also catalyzes the addition of pyruvate to cyclohexane-1,2-dione **95**, resulting in a similar conversion of **96** (25%) but also with a higher enantiomeric excess (*ee* = 88%) [109]. Regarding linear products, **94a** was obtained with 31% conversion, while for **94b,** a conversion of 24% was reported with an ee of 54% for the *S*-enantiomer. 

**Scheme 15:**
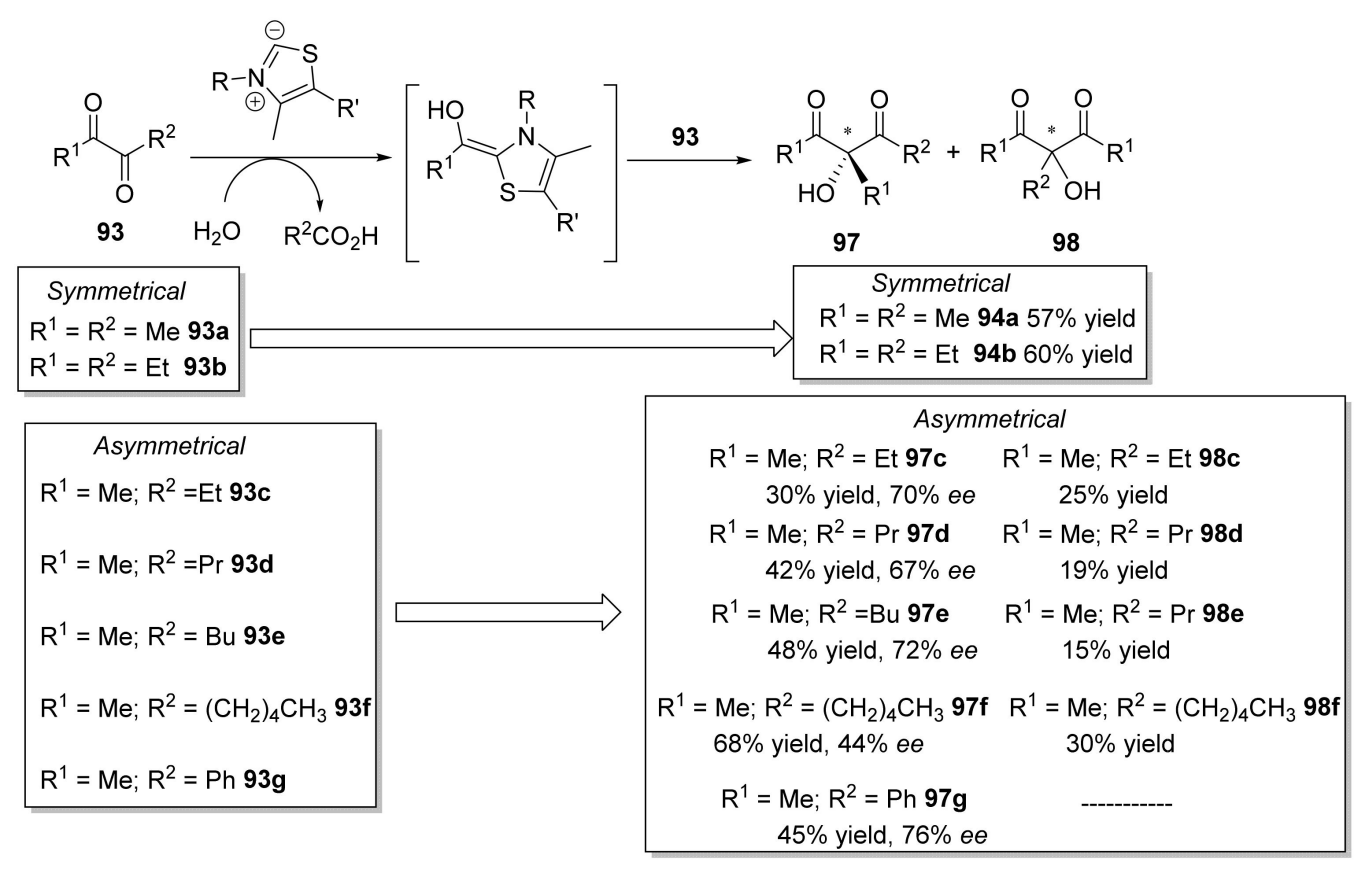
Preparation of symmetrical and asymmetrical 1,3-diketones by using acetylacetoin synthase.

Further, in 2010, the same year when Müller and coworkers reported the use of *Yp*YerE [107], Giovannini et al. described the use of an acetylacetoin synthase (contained in cell-free extracts of *Bacillus stearothermophilus* cultivated on acetoin as the main carbon source) for catalyzing a similar aldehyde-ketone cross-benzoin-type reaction (Scheme 15) [110]. This enzyme promoted the ThDP-dependent cleavage of α-diketones **93**, leading to the in situ formation of reactive aldehyde intermediates (acetyl and propionyl anion equivalents) and the release of the corresponding carboxylic acids. The subsequent attack of the acyl anion equivalent on one of the two carbonyl groups of the α-diketone **93** led to symmetrical achiral products (**94a** and **94b**), as previously reported with *Yp*YerE, or to a mixture of chiral products **97** (*R*-configuration) and achiral compounds **98**, in all cases with moderate yields and different ratios.

The optically active products **97**, some of which are known flavoring agents [111], always showed *R* configurations and *ee* values ranging from 67 to 91% [112]. This homocoupling of α-diketones has also been reported using continuous-flow conditions in a packed-bed reactor, in which the purified enzyme purified had been immobilized onto mesoporous silica [113]. Remarkably, Giovannini and coworkers reported [114] that the real enzyme responsible for reactions in Scheme 12, termed acetylacetoin synthase up to that moment, was, in fact, acetoin:dichlorophenolindophenol oxidoreductase (Ao:DCPIP OR), also used by the same group for catalyzing the asymmetric synthesis of phenyl acetyl carbinol (PAC) analogs [115]. Comparing the stereobias of this enzyme with that from *Yp*YerE, for open-chain ketones, the two enzymes afforded products (**94b**, **97**) with opposite stereochemistry.

An enzyme homologous (59% amino acid identity) to *Yp*YerE, *Pp*YerE from *Pseudomonas protegens* has been recently identified, crystallized, and its 3D structure has been determined [116]. Anyhow, comparing yields and *ee* values reported in the preparation of compounds **94b** and **96**, results with *Pp*YerE are not better than those obtained with *Yp*YerE.

### 3.2. Organocatalytic Methodologies for the Preparation 1,3-Diketones

**Scheme 16:**
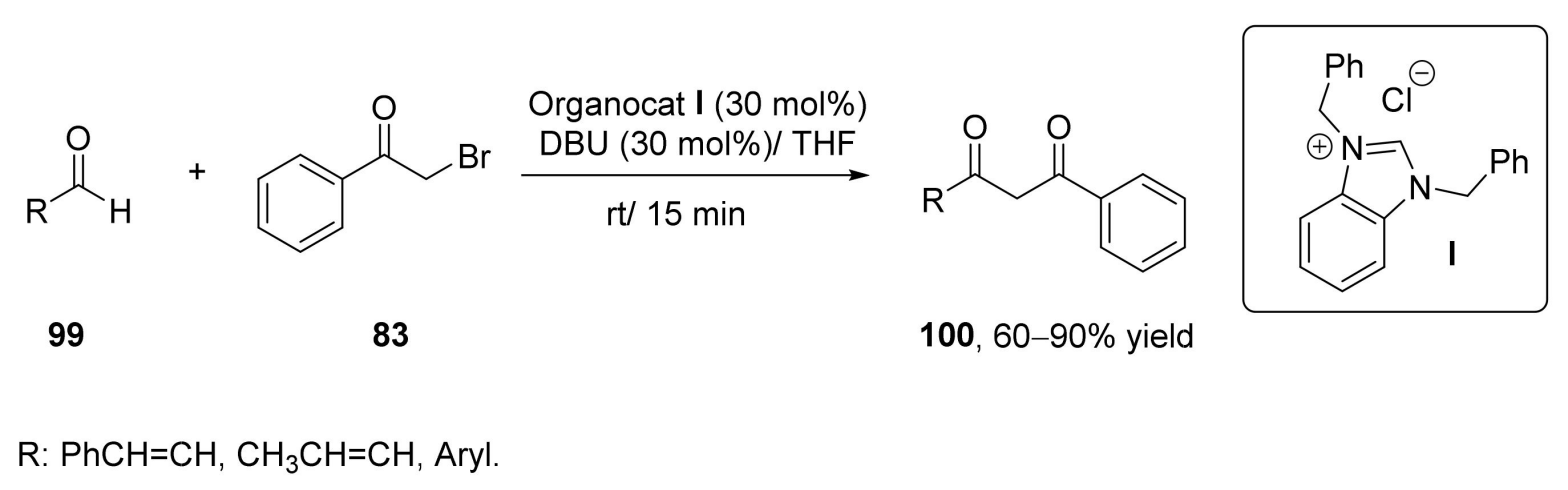
Organocatalyzed synthesis of 1,3-diketones employing *N*-heterocyclic carbenes.

The use of relatively small organic molecules as catalysts in organic reactions has gained an overwhelming interest during this century [117,118]. This catalytic approach has also been employed for the preparation of 1,3-diketones. Among the organocatalysts, *N*-heterocyclic carbenes (NHCs) have been used as catalysts in different organic reactions [119], including nucleophilic substitution reactions. It was reported that NHCs were able to participate in the intermolecular nucleophilic acylation of benzyl halides with aldehydes, but no examples of the acylation of α-haloketones via acyl anion equivalents of aldehydes and enals were shown until 2011, yielding to 1,3-diketones and α,β-unsaturated 1,3-diketones [120], as indicated in Scheme 16. Thus, different types of NHCs (30 mol%) were studied in the reaction between benzaldehyde or cinnamaldehye (**99a**; R = Ph–CH=CH) with phenacyl bromide (**83**) for preparing the corresponding 1,3-diketones **100**. Carbene **I** afforded the highest conversions in the preparation of both compounds, with yields around 85% when employing 1,8-diazabicycloundec-7-ene (DBU) as a base, and the reaction was conduction in THF as solvent at 30 °C (Scheme 16). These reaction conditions were extended to the preparation of other aromatic 1,3-diketones with yields from good to excellent (60–90%), whereas the use of aliphatic aldehydes or α-haloketones led to low yields (15–25%).

### 3.3. Synthesis of 1,3-Diketones Using Metal-Based Catalysts

**Scheme 17:**
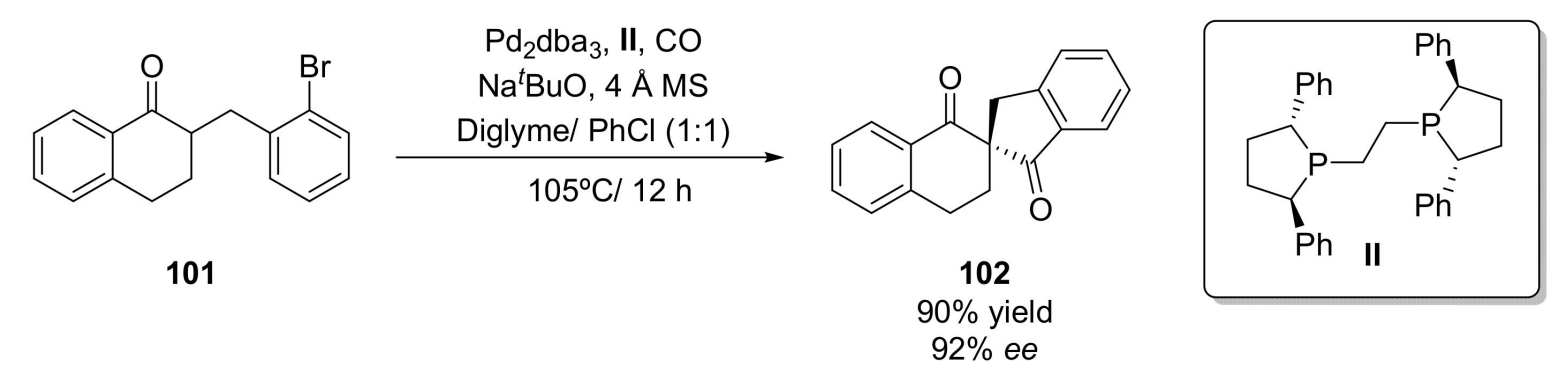
Metal-based catalytic preparation 1,3-diketones in presence of a palladium complex.

Metal catalysts have also been employed for the preparation of 1,3-diketones. One example has been recently described for the preparation of chiral spirocyclic β,β’-diketones (**102**) [121] through a palladium-catalyzed α-carbonylative arylation. Thus, 2-(2-bromobenzyl)-3,4-dihydronaphthalen-1(2*H*)-one (**101**) was treated with CO at 105 °C in diglyme in the presence of Pd_2_dba_3_ (2.5 mol%) and a phosphorous ligand (**II**) using NaO*^t^*Bu as base as 4 Å MS as an additive (Scheme 17). The phosphorous ligand plays a key role in the process activity and selectivity; it was observed that the use of (*R*,*R*)-Ph-BPE, an ethylene bridged chiral biphospholane led to the desired diketone in high yield (92%) and optical purity (88% *ee*). The use of aromatic solvents led to an increase in the process selectivity, but lower yields were achieved. When a mixture diglyme/chlorobenzene 1:1 was employed as a reaction solvent, the final product was isolated in 90% yield and 92% *ee*. The reaction scope was analyzed, it was observed that a wide set of spirocyclic diketones with different sizes of rings, substituents, and functional groups were prepared with high yields (67–94%) and optical purities (71–96%), demonstrating the versatility of this transformation. The process was scaled up to grams in the synthesis of a difluorinated diketone in a multistep process from a starting bromoolefin. Under the optimized conditions, the chiral β,β’-diketone was recovered in 90% yield and 93% *ee*, which shows the practical application of this method.

## 4. Synthesis by α-Functionalization of Other 1,3-Diketones

The preparation of substituted 1,3-diketones can be performed from other simpler 1,3-diketones through different methodologies. In the last few years, some examples have been developed to carry out these synthetic procedures.

**Scheme 18:**
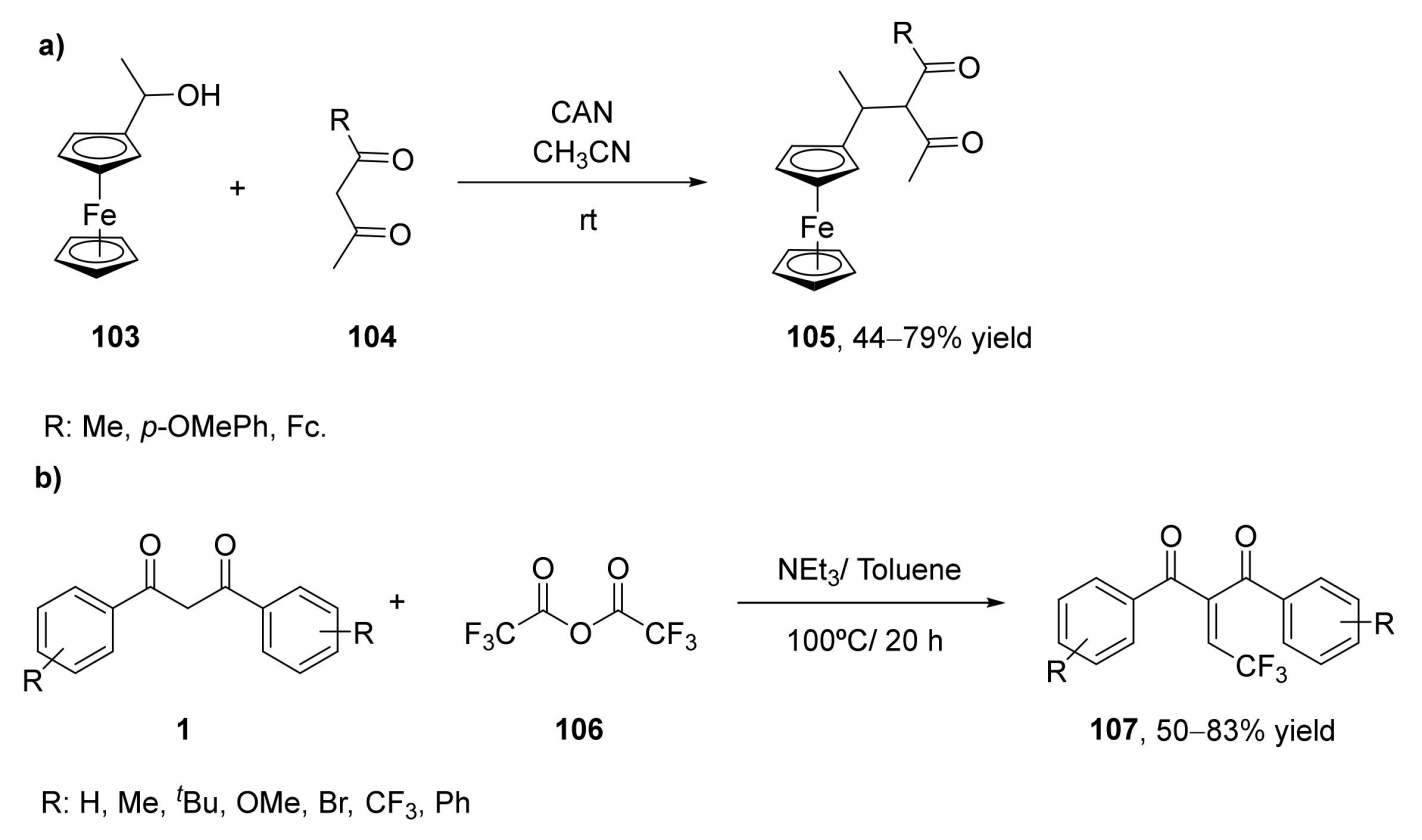
Synthesis of novel β-diketones by functionalization at the α-position of other 1,3-diketones: (**a**) preparation of chiral ferrocene-containing 1,3-diketones (**105**); (**b**) preparation of the preparation of 2-(2,2,2-trifluoroethylidene)- and (2,2-difluoroethyl)-1,3-dicarbonyl compounds (**107**).

Chiral ferrocene-containing 1,3-diketones (**105**) have been prepared from 1-ferrocenyl ethanol (**103**) with the corresponding 1,3-diketones (**104**) [122], as shown in Scheme 18a. The carbon–carbon bond formation was performed using cerium(IV) ammonium nitrate (CAN) at room temperature for 0.5 to 48 h [123]. Three 1,3-diketones have been obtained, containing the methyl, the 4-methoxyphenyl, and the ferrocenyl group in their structure, respectively. It has been observed that the size of the substituent presents high importance in the reaction development, as higher yields and shorter times were required for the smaller groups. Yields ranged from 79% for the methyl substituent to 44% for the ferrocenyl one. Solution and solid-state measurements have indicated that these three compounds exist only as diketo tautomers.

Calix[4]arenes and calix[4]resorcines have been recently employed as platforms for the linkage of different 1,3-diketones, in order to prepare compounds that could be used with biomedical applications as contrast agents due to their photophysical properties [124]. The synthetic procedure for the preparation of these diketones was carried out using halogen-methylated derivatives of calix[4]arenes for anchoring the dicarbonyl moiety through the addition of sodium salt of the corresponding diketone to the 5,7-bis-(bromomethyl)-25,26,27,28-tetrahydroxycalix[4]arene in anhydrous 1,4-dioxane as solvent. The final compounds were recovered with good yields (52–60%) even when working at room temperature.

In 2019 the preparation of valuable 2-(2,2,2-trifluoroethylidene)- and (2,2-difluoroethyl)-1,3-dicarbonyl compounds (**107**) through a Knoevenagel condensation was reported (Scheme 18b) [125]. This is an important reaction for the formation of C–C bonds leading to trisubstituted alkenes as final products. The reaction started from a 1,3-diketone, 1,3-diphenylpropane-1,3-dione (**1**), which was treated with trifluoroacetic anhydride (**106**) in the presence of different amines using toluene as solvent at 100 °C. The highest yield in the formation of the 2-(2,2,2-trifluoroethylidene)-1,3-dicarbonyl compound was achieved in the presence of triethylamine (NEt_3_) as a base while using 5.0 equivalents of anhydride and 7.0 equivalents of amine, leading to 95% of the desired product. Once optimized this reaction, the substrate scope was studied with other bisbenzyl 1,3-dicarbonyl compounds. In general, those symmetric compounds led to the final compounds with moderate to good yields. There is no steric effect on the phenyl ring of the starting materials, but it was observed an effect of the electronic nature of the phenyl ring substituents. Thus, those diketones bearing electron-withdrawing groups led to lower yields in slower processes. Heterocyclic diketones afforded the final products with high yields (around 80%), whereas those asymmetric starting materials led to good yields (72–85%). Once observed that a wide set of aromatic diketones can react, a partially fluorinated difluoroacetic anhydride was tested as a reagent in the condensation. The reaction afforded as final products the reductive products (alkanes) instead of the Knoevenagel alkenes, but yields were low (20–35%).

The reaction of 1,3-diphenylpropane-1,3-dione with trifluoroacetic anhydride was performed at multi-gram scale. Thus, 2.24 g of the diketone **1** (R = H) led to 2.52 g of the desired product after 20 h, with 83% of isolated yield.

## 5. Conclusions

The preparation of 1,3-diketones has traditionally been (and it still is) an area of the highest interest for organic chemists, as can be inferred considering the number and variety of research developed in this field, and commented in the present review. 1,3-Diketones present a high interest not only by themselves, but also as precursors of a wide range of valuable molecules, including several types of carbocycles and heterocycles. In fact, a plethora of therapeutical uses are associated with these compounds, as described in the introduction section.

It is well established that the most common method for the synthesis of 1,3-diketones involves the Claisen condensation between an ester and a ketone, a technique still nowadays widely employed for the preparation of novel compounds. This methodology can be performed with certain modifications, as exemplified by the use of soft enolates (carboxylic acids) for the synthesis of base-sensitive 1,3-diketones. Apart from the Claisen approach, several techniques have appeared for the synthesis of these valuable scaffolds. Thus, the hydration of alkynones or the decarboxylative coupling of carbonyl compounds represent two interesting approaches for preparing 1,3-diketones with moderate to good yields.

Special attention has to be given to the catalytic methods for the synthesis of 1,3-diketones, especially for those metal-free synthesis represented by the use of enzymes or organocatalysts in catalytic amounts for the enantioselective (when required) synthesis of optically active β-diketones.

Finally, the reaction of different electrophiles with the α-carbon atom of a β-diketone also represents a well-established method for the synthesis of novel α-substituted-β-diketones, still applied in recent years.

In summary, very attractive new methodologies have been developed during the last decade aiming to prepare novel valuable structures containing the 1,3-diketone scaffold with the highest yields. Anyhow, this is still a work in progress, as further improvements can be foreseen to be reported in the next future.

## Data Availability

Data sharing not applicable.
